# Optimization of an Elastic Network Augmented Coarse Grained Model to Study CCMV Capsid Deformation

**DOI:** 10.1371/journal.pone.0060582

**Published:** 2013-04-16

**Authors:** Christoph Globisch, Venkatramanan Krishnamani, Markus Deserno, Christine Peter

**Affiliations:** 1 Max Planck Institute for Polymer Research (MPIP), Mainz, Germany; 2 Department of Physics, Carnegie Mellon University, Pittsburgh, Pennsylvania, United States of America; German Cancer Research Center, Germany

## Abstract

The major protective coat of most viruses is a highly symmetric protein capsid that forms spontaneously from many copies of identical proteins. Structural and mechanical properties of such capsids, as well as their self-assembly process, have been studied experimentally and theoretically, including modeling efforts by computer simulations on various scales. Atomistic models include specific details of local protein binding but are limited in system size and accessible time, while coarse grained (CG) models do get access to longer time and length scales but often lack the specific local interactions. Multi-scale models aim at bridging this gap by systematically connecting different levels of resolution. Here, a CG model for CCMV (Cowpea Chlorotic Mottle Virus), a virus with an icosahedral shell of 180 identical protein monomers, is developed, where parameters are derived from atomistic simulations of capsid protein dimers in aqueous solution. In particular, a new method is introduced to combine the MARTINI CG model with a supportive elastic network based on structural fluctuations of individual monomers. In the parametrization process, both network connectivity and strength are optimized. This elastic-network optimized CG model, which solely relies on atomistic data of small units (dimers), is able to correctly predict inter-protein conformational flexibility and properties of larger capsid fragments of 20 and more subunits. Furthermore, it is shown that this CG model reproduces experimental (Atomic Force Microscopy) indentation measurements of the entire viral capsid. Thus it is shown that one obvious goal for hierarchical modeling, namely predicting mechanical properties of larger protein complexes from models that are carefully parametrized on elastic properties of smaller units, is achievable.

## Introduction

In the past three to four decades the field of Molecular Dynamics (MD) simulations has matured into an indispensable and well-established tool in bio-molecular science [1]–[10]. The rapid development and improved accuracy of force fields [11] has turned computer simulations into a reliable and, more importantly, an insightful method for describing and understanding biological phenomena [12]. Time and again, atomic level details obtained from these simulations have proven crucial in understanding bio-molecular function of complex biological systems [1]–[15].

In biology, the application of computer simulations spans an impressive range, all the way from sub-atomic resolution chemical changes in the active site of an enzyme [16]–[20] to single molecule level domain motions [21], [22] up to protein-protein associations [23] and assembly of large molecular complexes like actin [24] and microtubules [25].

However, bigger systems move more slowly. In fact, dispersion relations for dissipative soft matter are frequently of the form 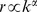 with *r* being a general relaxation rate, *k* the corresponding wave vector and 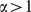 (e.g. 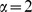 for diffusion or 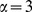 for membrane undulations), implying that the time problem is often the more severe one, and it is much less amenable to parallelization techniques such as domain decomposition or running different replicas. Tremendous computational effort and/or simulation time are required for studying large biological ensembles such as ribosomes, multi-protein assemblies such as viruses and biological processes like protein (un-)folding [26], [27], protein self-aggregation etc., in a biologically relevant time scale in atomistic resolution.

One promising way to overcome these limitations is coarse graining (CG). As early as 1975 Levitt and Warshel have suggested to tackle the protein folding problem by reverting to a reduced set of relevant slow variables, relegating all finer detail to averages [28]. Hagler and Honig [29] subsequently pointed out that without stringently defined criteria for measuring folding success, a wide spectrum of plausible structures might superficially look good; but recent developments of systematic coarse graining methodologies [30]–[33] seem to provide a clean framework for addressing many possible concerns and have given the field new momentum.

Nevertheless, any CG model by construction leaves out certain aspects of the small-scale physics, causing possible problems on larger scales one must either tolerate or fix. For instance, the MARTINI protein model [34], [35] represents three to four heavy atoms by a single CG bead, and for the peptide backbone this leaves one isotropic CG bead per amino acid; this resolution is too low to permit secondary structure formation, let alone folding. The developers of MARTINI hence propose to stabilize native protein structures by introducing extra harmonic bonds of suitable strength between selected degrees of freedom. This of course disqualifies the model for protein folding studies, but protein partitioning between different phases [35] or assembly of protein complexes [36], [37] are well within its reach, especially since partitioning coefficients are a central target that MARTINI’s CG interactions aim to reproduce. In this article we investigate the question, how such extra stabilizing networks should be constructed to capture not only single-protein fluctuations but also the flexibility of larger assemblies, thereby expanding on earlier work focusing on single proteins [38]. We will show that this obvious goal for hierarchical modeling, namely predicting elastic aspects of larger protein complexes from models that are carefully parametrized on smaller units, is achievable.

The idea to add a stabilizing network to a CG simulation goes back to elastic network models, which in turn were strongly influenced by Paul Flory’s work on polymers [39]. Tirion showed in 1996 [40] that the frequency spectrum of a protein is remarkably well reproduced by the following crude model: link all atom-pairs in a protein structure within a pre-specified cutoff with harmonic springs of a rest length equal to the atom distance and always the same spring constant *K*. A slightly different setup (harmonic in the pair vectors) was proposed by Bahar et al. [41]. Elastic network models (ENM) showed that the linear response of proteins (equilibrium fluctuations and correlation times) is essentially contained in the static crystal structure [40]–[47]. Running a simulation is neither necessary for fixing the input (one spring constant rules all scales) nor for arriving at the output (harmonic systems can be solved analytically). Both of this changes, though, if we are interested in physics beyond the linear regime – in the present case, if we add a stabilizing harmonic network on top of the generally very nonlinear interactions which together constitute a (CG) protein model: The energy scale is set and the spring constant(s) must be chosen relative to the existing force field, which ideally already captures part of the interesting physics. Since only very few nonlinear systems can be solved analytically, one is usually forced to apply numerical approximation methods. Hence the resulting hybrid system (force field plus elastic network) has to be simulated.

In this paper we investigate some of the intricacies that arise, when an artificial network is added on top of a CG model to amend some of its shortcomings. For instance, the classical cutoff criterion for adding springs – according to which two neighboring units closer than a pre-specified cutoff are linked – can lead to the placement of erroneous bonds, simply because whatever served as a target structure coincidentally showed a close distance, while the separation of these two beads in fact fluctuates widely over time. Or, proteins with identical amino acid sequence might assume slightly different folded structures, according to their location in a bigger complex. The capsid subunits of CCMV, which all have the same amino acid sequence but assume three slightly different folds, depending on which of the three symmetrically inequivalent sites on the capsid they assume, are to some extent an example of such a case. The majority of these differences in the fold are localized to the N- and C-termini. While the former interacts with the RNA and is not relevant to the present study (see below for details), the latter is a dimerization motif. As a consequence, the C-terminal structural differences between different protein monomers are intimately linked to the overall shape of the protein dimers. Clearly, these differences are not innate to the capsid monomer but result from its incorporation into the overall structure. Though the RMSD differences between the different chains are small within the protein core, overall they would result in three different CG models. And it seems ill-advised to engage in aggregation studies by *starting* with three inequivalent reference structures – and consequently inequivalent CG models for the monomers. In fact, parts of the chain might only assume a well-defined structure *after* they have aggregated. The C-terminal tail of the CCMV capsid monomer is an example for this case: it is largely extended and unlikely to be stable in its specific conformation in a single monomer/capsomer, but fits extremely well into grooves on a second one, thus leading to formation of a stable dimer. (Note that from now on we will be using the terms “capsid protein”, “capsid monomer” and “capsomer” equivalently, all of which refer to a single subunit of the viral capsid.) Unfortunately, it is difficult to avoid the above-mentioned biases while relying on experimental structure data alone, because our high resolution knowledge of a capsid protein’s crystal structure precisely derives from studies of full capsids, and the immense efficiency with which these proteins aggregate into capsids prevents us from crystallizing them into anything else that would avoid the capsid-derived bias. However, based on the experimental structures one can perform atomistic simulations to obtain the missing information about such structural fluctuations. We used the systematic refining of the elastic networks as described by Lyman et al. [48] to introduce the “missing” fluctuation modes in the coarse grained model. One can simulate monomers, dimers, or larger capsid fragments and study how their large scale behavior emerges. In other words, atomistic reference trajectories of different assembly stages constitute a hierarchical sequence that can be used as a guiding principle for constructing elastic-network fortified CG models. This is the strategy we follow in this work.

### System

**Figure 1:**
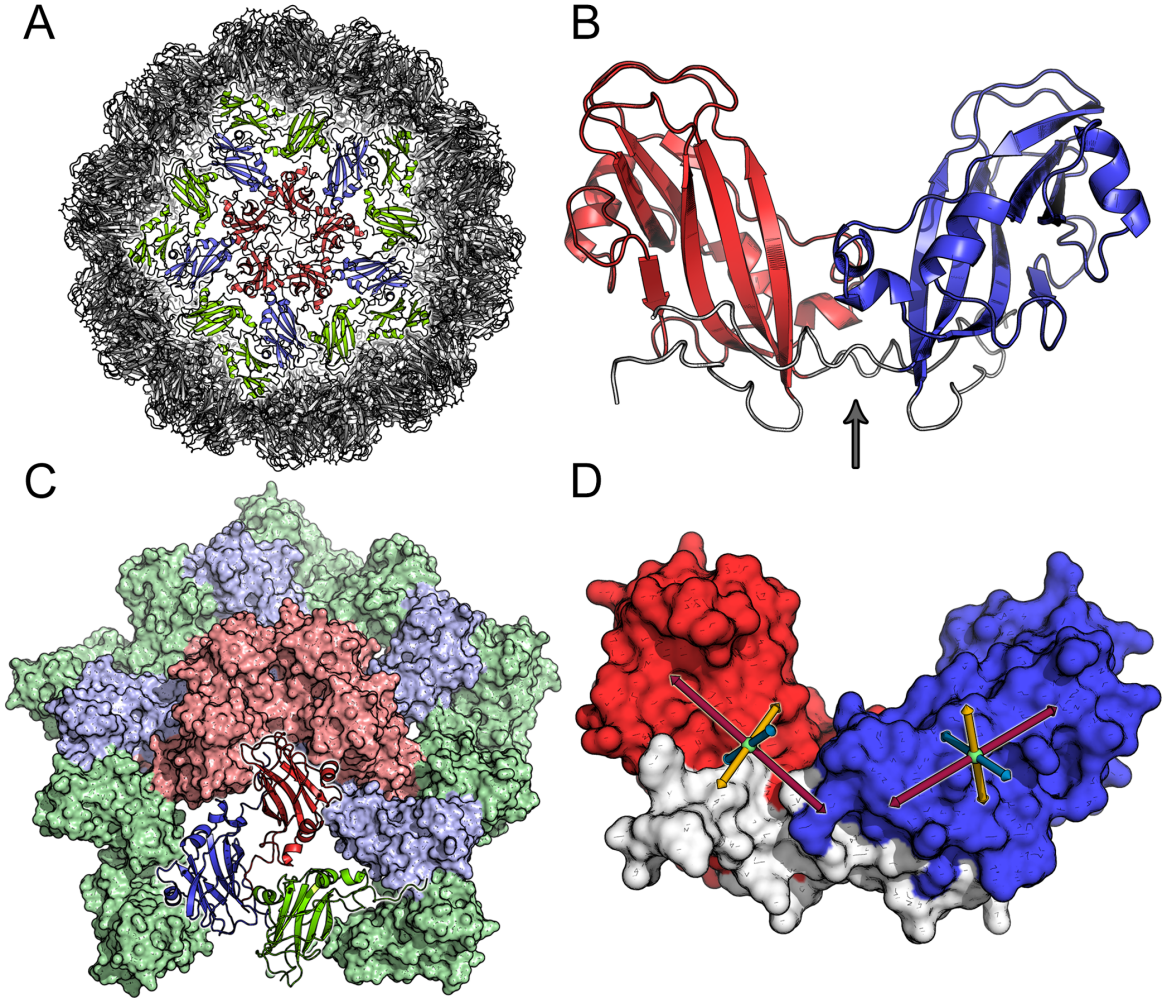
Different representations of the CCMV capsid and its subunits. (A) Render of the whole CCMV viral capsid, highlighting pentamer of dimers (POD) (red and blue) plus the flanking CC dimers (green). The type A chains in the POD are colored in red, while the type B chains are colored in blue. (B) Cartoon representation of the core regions for a dimer (residue 14 to 142 for each chain - without the flexible tails). This figure draws emphasis to the “hinge” (arrow points to the region) between the two capsomers, that enables them to rotate relative to each other. (C) Pentamer of dimers (red and blue) plus flanking CC dimers (green) highlighting the asymmetric unit (bottom in cartoon representation). (D) The defined internal axes (which are loosely based on the gyration tensor of the capsomer) for generating the relative orientation maps (see text). The center of mass (COM) of C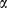 atoms of residues 69–71, 92 and 122–124 defines the ***X***-axis (red), the COM of the C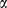 atoms of residues 20–21 and 134–135 defines the ***Y***-axis (yellow) and the COM of the C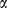 atoms of residues 56–58 and 99–100 defines the ***Z***-axis (blue).

Let us briefly describe the viral model system we investigate in this work. CCMV belongs to the Bromoviridae family of viruses. Its capsid has icosahedral symmetry and a triangulation number (T-number) of 3 (**Figure 1A**), using the terminology of `quasi equivalence theory’ as introduced by Casper and Klug [49]. The whole virus, as well as several structurally consequential mutants, have been crystallized [50]. The capsid consists of 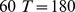 chemically identical capsomers, each consisting of 190 amino acids. A capsomer is folded into an 8-strand 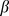-sheet core with the N-terminus extending into the interior of the capsid and the C-terminal tails making inter-capsomer contacts to form dimers (see **Figure 1B**). The capsomers self-assemble into an icosahedrally symmetric virus particle via several intermediates. In solution dimers form the predominant stable structure [51]. Experimental studies suggest that pentamers of dimers (POD) form an intermediate seed complex for building the whole capsid by further addition of dimers. [51]–[53].

In-vitro experiments have shown that deletion mutation of the N-terminus (1–36 residues) prevents the packaging of RNA but does not disrupt the formation of the capsid [52], [53]. In this study, we shall be using this Δ1–36 mutant capsomer protein in all our simulations. Throughout the paper we will use the residue numbering of the Δ1–36 mutant, this means that residues 37, 38, …, 180 of the full capsomer sequence will be referred to as residues 1, 2, … 154.

By virtue of the location of the capsomers at the various symmetry centers of the icosahedron, they adopt 3 marginally different structural conformations (A, B and C) (see **Figure 1C** and **Table S1** in **File S1**). The pentameric association site forces the capsomers to assume conformation A, whereas the hexameric association site enforces conformations B and C, forming a quasi-six-fold rotational symmetry of the hexamer. The majority of these structural differences are localized to the N- and C-terminal regions of the protein chain (see **Figure S1** in **File S1**). The N-terminal tails form a 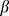-barrel core at the hexameric association site, whereas there is no evidence of organized structure at the pentamer interface. We have recently argued that this can indeed be understood as a collective geometry-dependent impact on local structure formation during the assembly process [54] Note however, that in the Δ1–36 mutant, the N-terminal differences between A, B, and C folds are largely cleaved away, i.e. not relevant to the present study. The C-terminal structural differences between different protein monomers, however, are intimately linked to the overall shape of capsomer dimers (see **Figure S2** and **Table S2** in **File S1**). For dimer formation two protein chains interact via their respective C-terminal tails which form a flexible linker region. Since these dimers are the smallest units proposed for the assembly process, and since the overall shape of the dimer is influenced by these small conformational variations in the C-terminal interface regions, special attention has to be paid to the representation of these regions in the CG model.

Summarizing, the presence of a high-resolution structure of CCMV, the ability of capsomers to self-assemble without active biochemical control, the availability of various in-vitro studies, and a host of intriguing unresolved questions (such as the back-effect of global structure on local folding of capsomers) establish the capsid of CCMV as an attractive model system for the type of questions we wish to address.

## Results

A key aim of our work is to test how the different prescriptions for setting up a supporting elastic network affect the local fluctuation spectrum of monomers and dimers, how these fluctuations change in larger capsid fragments, and what impact they have on the large-scale elasticity of the whole viral capsid. We performed atomistic simulations of several capsomer complexes. In the present paper we focus on dimers and a system of pentamers of dimers with an additional ring of CC-dimers (POD+CC) (i.e. a highly symmetric capsid fragment consisting of 20 monomers, see **Figure 1C**). While the dimer is the main reference system for the CG model for which the parameters are derived, the POD+CC system is used to test the agreement between the atomistic and the CG simulations. It serves as a model complex, where atomistic reference data can still be obtained – albeit with considerable computational effort – and where one can verify that a CG model, which was parameterized based on fluctuation data of individual dimers in solution, can indeed correctly reproduce the behavior of the same dimers, when they are placed in a capsid-like environment. The POD+CC unit was chosen since the pentameric unit is experimentally proposed to be important for structure and assembly of the capsid, but – more importantly – because of its very high symmetry and the two characteristic types of dimers it is composed of: the center of the POD+CC complex is formed by five equivalent AB dimers which are completely surrounded by other proteins, potentially restricting the dynamics of these dimers. In contrast, the five equivalent outer CC dimers are more exposed to the solvent and possibly less restricted by the rest of the complex (**Figure 1C**).

In a last step, we will study entire CCMV capsids with CG simulations and show how in different CG models (i.e. different elastic networks) the parameters of individual capsid proteins influence the elastic response of the entire capsid. We will evaluate the elastic networks by setting up comparable indentation simulations to correspond with experimental mechanical deformation studies. This hierarchy of scales and model systems will permit us to evaluate the necessary level of detail one needs to invest to obtain a consistent set of structures with correct elastic properties.

### Atomistic Simulations of Dimers

The atomistic simulations are on the one hand used to characterize the relevant motions in the complexes, in particular to find out how the dynamics within the individual proteins and the relative motion of proteins within dimers and larger complexes are affected by the presence or lack of protein environment. On the other hand, these simulations will serve as a basis for the setup and the refinement of the supporting elastic network in the coarse grained model.

Three independent atomistic simulations (100 ns each, with a different random seed for the initial velocities) were performed with the aim to sample the relative conformational flexibility of the dimers. One of these simulations was additionally extended to 400 ns.

**Figure 2:**
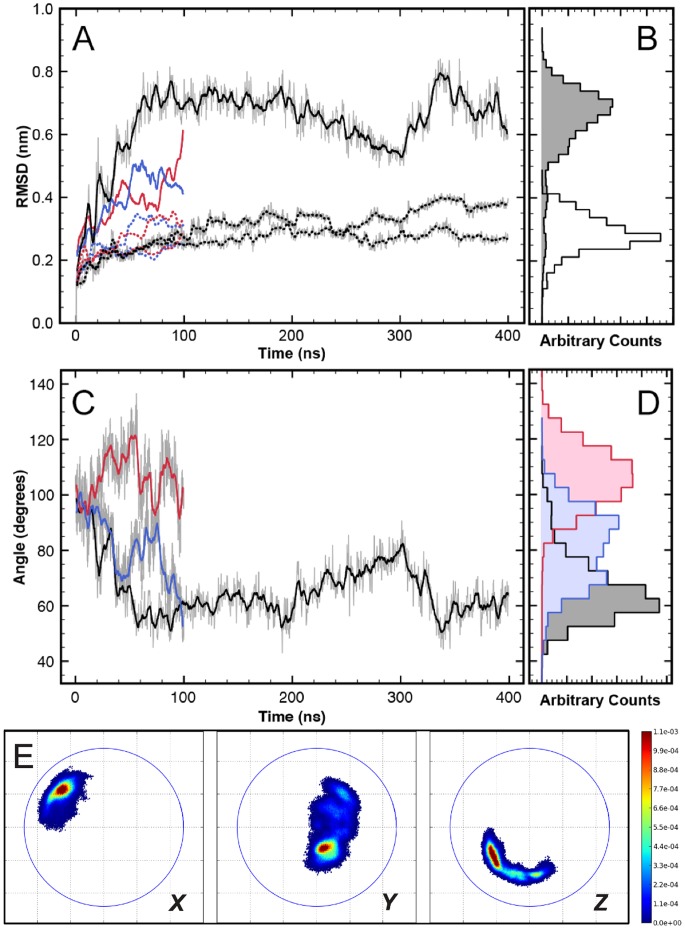
Atomistic MD simulations of the free dimer in solution. Colors black, red and blue represent different independent simulations. (A) Time series of RMSD of core regions (see Materials and Methods) 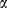-carbons in the capsomers (dotted) and the dimer (solid). (B) Histogram of RMSDs sampled by the capsomers (unshaded) and dimer (shaded) for the 400 ns simulation. (C) Time series of relative “twist angle” of capsomers in the dimer (see also **Figure S3** in **File S1**). (D) The corresponding “twist angle” distribution for the three simulation trajectories. (E) Relative orientation maps (ROMs) of the dimers (averaged over all atomistic simulations, i.e. a total of 600 ns). For details regarding the projections shown see main text. Coloring according to the normalized probability of finding these relative orientations. The blue circles are drawn with a radius of the longest internal axis of the capsomer.

The structural stability of the proteins is analyzed via the root mean square deviation (RMSD) value of the 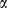-carbons from the initial/experimental conformation (**Figure 2A, B**). Here, the RMSD values of the two individual capsid proteins (dotted lines) characterize the internal flexibility of the proteins. The comparatively low average RMSD values of 2–3 Å indicate that the capsomers are structurally relatively stable. **Figure 2A, B** also shows the RMSD of the entire dimer (solid lines), which is substantially larger since it also captures the overall deformation of the protein complex, including the large-scale relative motion that is mostly happening due to the flexible “hinge”, where the proteins are linked via their C-terminal tails (see **Figure 1B**). This “hinge” motion will be analyzed in greater detail below. Although time series are convenient in visualizing meta-stable states of the conformations, for the sake of lucidity and succinctness we choose to use histograms (**Figure 2B**) for subsequent analysis and comparison with the CG simulations. These meta-stable states (we discuss examples below) tend to show up as multiple peaks (“multi modality”) of the histogram distributions.

The relative orientation of the two capsomers in the dimer complex can be described by various angles between vectors defined within the individual proteins. **Figure 2C**, for example shows the time evolution of an angle that characterizes a “twist” motion which is defined by planes spanned within the proteins (for an illustration see **Figure S3** in **File S1**). The time evolution in the different independent dimer simulations shows that this twist angle can “get locked” in different values and remains there for the entire length of the simulation (**Figure 2C**). Extending the simulation further to 400 ns does not seem to improve the sampling of other conformations once the twist orientation gets locked. The angles sampled by the dimer in the different simulations span a range of 70 degrees (**Figure 2D**). Such large variations and preferential orientation “locks” of the dimer in separate simulations suggest that sampling of large scale structural fluctuations in this protein dimer is beyond the timescale of these atomistic simulations. This observation alone strongly points towards the need for coarse-grained simulations in enabling access to longer time scales and thus achieving better conformational sampling. One problem here is of course that even long and multiple atomistic simulations of the dimers will not allow us to extensively sample and equilibrate the dimer dynamics and the hinge motion. We conclude that the parametrization of the CG model can only be based on shorter-timescale dynamics such as fluctuations within the individual monomers. Nevertheless, the extent of the relative protein orientations within the dimers observed in the atomistic simulations will be analyzed further in the next paragraph, since this will provide us with information regarding the mobility at the “hinge”. Due to its connection with the shape and deformability of the dimers, this “hinge” motion will be important both for assembly and mechanical properties of the capsids and is therefore an important criterion for the CG model.

Descriptors such as a single angle are hardly sufficient to provide a good characterization of the three dimensional arrangement of two anisotropic units (in this case capsomers) in a dimer connected by a hinge. Therefore a better representation that describes the relative orientations between the two monomers has been developed in form of relative orientation maps (ROMs) of all principal axes of the capsomers (described in detail in the Methods section). Briefly, a ROM stereo-graphically projects a principle axis of one capsomer onto an equatorial plane whose normal vector is the corresponding principal axis of the other capsomer. Relative positions of the capsomer in the dimeric complex are plotted as probability densities as shown in **Figure 2E**. They quantify the relative orientation fluctuations of the capsomers by separately looking at the (***X*** *,* ***Y*** and ***Z***) ROMs. The ROMs of internal ***Y***- and ***Z***-axes describe a “twisting”-like rotation of the capsomers with respect to each other, whereas the ***X***-axis describes an “opening/bending” motion. **Figure 2E** shows as an example the three ROMs obtained from an average over all atomistic trajectories of the free dimer, i.e. a total of 600 ns of simulation time. Among the various regions visited during these simulations there are some favorable states with higher occupancies. After studying these ROMs for several atomistic and CG trajectories, we found that they are well suited to comprehensively describe the phase space sampled by the protein dimers and the extent of the relative motion around the hinge. The spread of the distributions of these relative orientations provides a very good visual guide and we will use this in the following to assess the dimer flexibility in different simulations and to compare simulation models. In addition we also provide means and standard deviations of the orientational distributions in the Supplementary Material (see **Table S4**, **S5** and **Figure S10** in **File S1**).

### Coarse-grained Simulations with Standard MARTINI Elastic Network

We first performed coarse-grained MD simulations using the MARTINI CG forcefield for proteins [34], [35], extended by the standard ELNEDYN network [38].

As a consequence of the slightly different structures assumed by the A, B, and C protein chains in the X-ray structure of the capsids (see **Figure 1C** and **Figure S1** in **File S1**), the application of the standard ELNEDYN network definition results in three slightly different elastic networks. It should be noted here that the elastic network is only established within each capsid monomer, there are no network bonds between different protein chains, and the intermolecular (protein-protein) interactions are purely handled through the non-bonded interactions of the MARTINI forcefield (i.e. van-der-Waals forces and Coulomb interactions). As one would expect one finds different elastic network bonds in the tail regions of the protein. This ambiguity in the elastic network definition based on the three reference structures illustrates the fundamental problem of how to deal with potentially flexible tails based on structure information only and opens up the question: how does one decide which elastic bonds should be applied and which should better be avoided since they would otherwise artificially restrict potentially flexible units?

**Figure 3:**
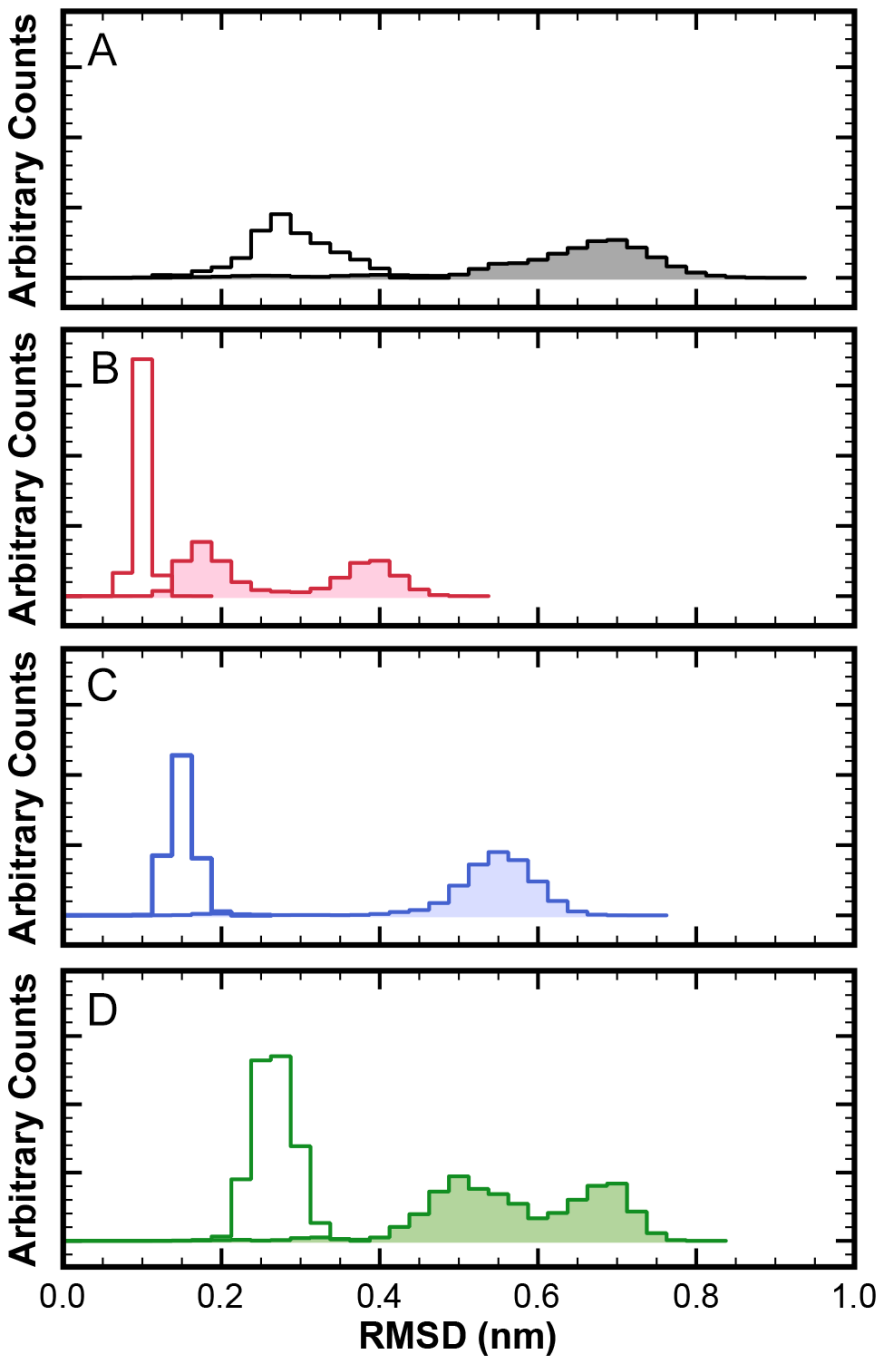
RMSD (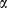-carbons of core regions) distributions from simulations of isolated dimers. Open histograms: RMSDs within the monomers; shaded histograms: RMSDs of dimers. (A) atomistic simulation (400 ns); (B) CG simulation with ELNEDYN network (spring constant: 500 kJ mol^−1^nm^−2^); (C) CG simulation with ELNEDYN network (spring constant: 200 kJ mol^−1^nm^−2^); (D) CG simulation with IDEN elastic network.

In a first CG setup, a uniform spring constant of strength 500 kJ mol^−1^nm^−2^ is used for all network bonds– in accordance with the standard MARTINI/ELNEDYN settings. The dimer is simulated for 400 ns (which corresponds to an estimated 1600 ns* in “real time” after mapping the accelerated CG dynamics to realistic timescales by a time-scaling factor of 4 that had been determined for the MARTINI force field [55], [56], see Methods section). The RMSD distributions in **Figure 3B** shows that the combination of this (monomer) elastic network with the MARTINI forcefield yields a stable dimer complex. However, the structural fluctuations of the capsomer (open distribution) and the relative motions between capsomers in the dimer (shaded distribution) are much too small, i.e. the protein complex is too rigid. (**Table 1** provides the respective means and variances of all RMSD distributions shown in **Figure 3** as a more quantitative analysis.) The bimodal nature of the dimer RMSDs reflects that the dimer adopted two meta-stable states, with a long-lasting persistence time of approximately 200 ns (time series not shown). Since this network made the proteins too rigid, the spring constant of the homogeneous elastic network was reduced by more than a factor of 2 to 200 kJ mol^−1^nm^−2^ (500 ns simulation time). **Figure 3C** shows that, interestingly, this partially “fixes” the deviations in the flexibility of the dimers (shaded distribution) and the RMSD values were much closer to those observed in atomistic simulations. However, the individual monomers (open distribution) are still too rigid. In the interest of being consistent, in all following figures data obtained from trajectory(ies) of atomistic simulation will be colored black, CG data from MARTINI plus ELNEDYN with spring constant of 500 kJ mol^−1^nm^−2^ will be colored red and from MARTINI plus ELNEDYN with spring constant of 200 kJ mol^−1^nm^−2^ blue. This coloring scheme should not be confused with the scheme used in **Figure 1** for highlighting the classification of dimers.

**Table 1:** Statistics of RMSD distributions of monomers and dimers.

	Dimers		Monomers	
2–3 5–6	*Mean* (*nm*)	*Std.Dev.* (*nm*)	*Mean* (*nm*)	*Std.Dev.* (*nm*)
**Atomistic**				
*Dimer*	0.6388	0.1168	0.2867	0.0546
*POD+CC* *Inner Dimer*	0.3962	0.1156	0.2029	0.0404
*POD+CC* *Outer Dimer*	0.4445	0.1188	0.2265	0.0482
**ELNEDYN** (*K*500)				
*Dimer*	0.2749	0.1059	0.0989	0.0088
*POD+CC* *Inner Dimer*	0.1812	0.0453	0.1096	0.0088
*POD+CC* *Outer Dimer*	0.2369	0.0384	0.1161	0.0124
**ELNEDYN** (*K*200)				
*Dimer*	0.5415	0.0667	0.1504	0.0156
*POD+CC* *Inner Dimer*	0.2568	0.0598	0.1575	0.0143
*POD+CC* *Outer Dimer*	0.2781	0.0660	0.1630	0.0170
**IDEN Network**				
*Dimer*	0.5765	0.0994	0.2665	0.0254
*POD+CC* *Inner Dimer*	0.3500	0.0432	0.2621	0.0300
*POD+CC* *Outer Dimer*	0.4528	0.0941	0.2695	0.0293

Data obtained from simulations of the free dimer (**Figure 3**) and the POD+CC complex (**Figure 6**), using the atomistic model, the CG model with ELNEDYN network with a uniform elastic network constant of 500 or 200 kJ mol^−1^nm^−2^ or the IDEN elastic network.

**Figure 4:**
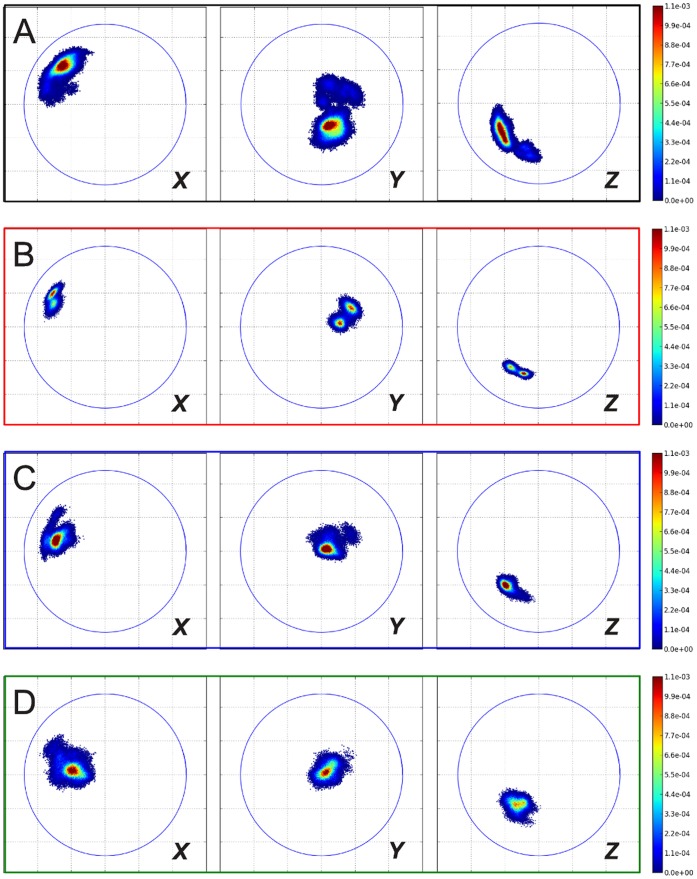
Relative orientation maps from simulations of isolated dimers. (A) atomistic simulation (400 ns); (B) CG simulation with ELNEDYN network (spring constant: 500 kJ mol^−1^nm^−2^); (C) CG simulation with ELNEDYN network (spring constant: 200 kJ mol^−1^nm^−2^); (D) CG simulation with IDEN elastic network. Projections of the ***X*** *(left panels),* ***Y*** *(middle panels),* ***Z*** *(right panels)*-axis of capsomer 2 on the *xy* plane when capsomer 1 is aligned to the *z* axis (see Methods section). Coloring according to the normalized probability of finding these relative orientations. The blue circles are drawn with a radius of the longest internal axis of the capsomer.

**Figure 4** shows the ROM analysis of the simulations with the two ELNEDYN networks (500 and 200 kJ mol^−1^nm^−2^). It clearly conveys that the sampling of the phase space by the dimer is greatly reduced by these networks compared to the atomistic simulations. Thus, making the individual monomers too rigid affects the *inter*-capsomer mobility and this calls for an effort to carefully refine the elastic network.

This also raises further questions: (a) Can a more refined elastic network capture the dimer behavior as observed in the atomistic simulations? (b) Can a uniformly refined elastic network capture the different behavior at the symmetrically inequivalent A, B, and C sites? (c) What is the biological relevance of the apparently vast relative orientational flexibility of the capsomers in a dimer during the viral capsid assembly process and for the mechanical properties of the resulting assembled capsid?

### Coarse Grained Simulations with IDEN Elastic Network

**Figure 5:**
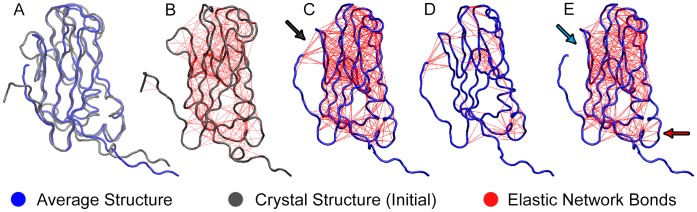
Illustration of the difference between the Elastic Networks. (A) Superposition of the initial structure (gray) of the atomistic MD simulation and the average structure (blue). (B) ELNEDYN network based on the crystal structure/initial structure of capsomer protein A from the PDB entry 1ZA7. (C) First step of the IDEN elastic network definition using just the distance criterion. When the distance criteria alone is used for elastic network definition it gives rise to artifacts such as the constraining of the flexible N-terminal tail (region pointed to by arrow) (D) The bonds that are removed from the network definition from (C) according to flexibility and concerned motion using the IDEN definition. (E) Final IDEN elastic network. The *cyan* arrow points to the unconnected N-terminal tail and the *red* arrow points to a loosely connected loop region by comparison to the network shown in (C).

We attempted to answer the above questions by constructing a refined universal elastic network to constrain the local flexibility, as described above. The following adaptations to the standard ELNEDYN network proved to be relevant: the bond definitions of ELNEDYN are based on the initial structure and ELNEDYN only uses a distance cutoff criterion. This has led to several unphysical/unnecessary bonds in the flexible regions of the protein (**Figure 5**), which artificially stiffened the capsomer. We saw evidence of this in the previous section when monitoring the RMSD of a capsomer and its dimer in our ELNEDYN coarse-grained simulations (**Figure 3** and **4**). In the IDEN (Iteratively-refined Distance-based Elastic Network) approach we define the bonds based on the distances between the atom pairs averaged over the two monomers in 400 ns of atomistic dimer simulations. This may exacerbate artifacts especially in regions with high mobility/fluctuations (the atoms might be in unphysical positions like in an average structure), as illustrated in **Figure 5C**. The flexible N-terminal tail is connected to the relatively rigid core of the capsid protein and would hinder free motion of this region. To avoid placing such artificial bonds we introduce extra checks (correlated motions and the extent of local structural fluctuations), and this takes care of grouping regions that move collectively on the one hand while preventing restricting mobile regions via unphysical springs on the other hand (**Figure 5D and E**). Further, IDEN scales the bond strength by iterative adjustment to account for domains that are relatively flexible. Note again that while the atomistic reference simulations used here are simulations of a dimer, also in the IDEN network derived form these simulations elastic network bonds are drawn only *within* a monomer, all CG inter-protein interactions are taken care of by the MARTINI forcefield.

**Figures 3D** and **Figure 4D** show the monitored descriptors (histogram of RMSD values and ROM, respectively) for 400 ns of CG simulations with our converged heterogeneous network (IDEN, in green). When compared with distributions obtained from ELNEDYN networks (**Figure 3B, C**), the IDEN-derived RMSDs of capsomers (open) and dimer (shaded) are in better agreement with the atomistic simulation. The dimer distribution using the IDEN network shows two meta-stable states as interpreted by the bi-modality of the distribution. The ROM projection spread in **Figure 4D** is now comparable in terms of the area swept by the conformations relative to one another. As opposed to the two ELNEDYN networks shown in the previous section, it is now possible to obtain a good agreement with the atomistic reference simulations both for the intra capsomer stiffness as well as for the extent of dimer motion at the “hinge”. Note that the comparison of the orientational distributions in the free dimer simulations between atomistic and CG simulations should no be over-interpreted since – as we have shown above – these large scale dynamics is by no means exhaustively sampled in the atomistic simulation. The conformationally more restrictive system studied in the subsequent section will be better suitable for a more quantitative assessment. At this point we would like to conclude again that elastic modes of individual capsomers (dictated by the elastic network) have a profound effect on the *inter*-capsomer mobility in a dimer.

This guides us to the following questions. (a) How does a careful parameterization of ENMs based on the structural fluctuations/the elastic modes of a “free capsomer” affect the properties of higher order aggregates? (b) What is the effect of the environment on a capsomer? We set out to address the above questions by simulating higher order aggregates in atomistic resolution and CG using MARTINI plus ELNEDYN and IDEN ENMs.

### Coarse Grained and Atomistic Simulations of Larger Capsid Fragments

**Figure 6:**
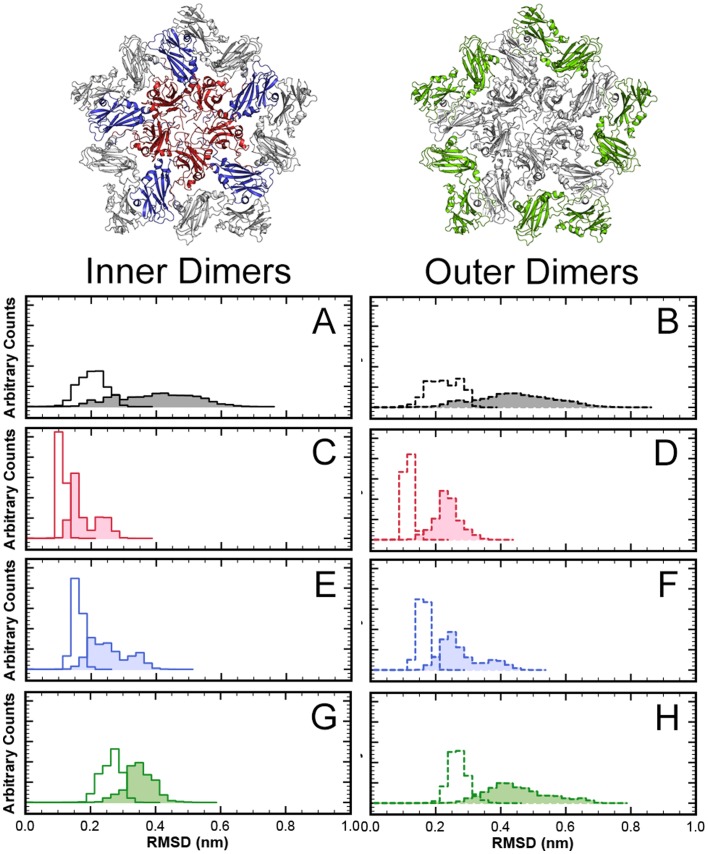
RMSD (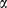-carbons of core regions) distributions from simulations of dimers within a larger capsid fragment. Pentamer of AB dimers with a ring of CC-dimers (POD+CC). Left side: Inner dimers of AB type (indicated in red and blue); Right side: outer dimers of CC type (indicated in green). Open histograms: RMSDs within the monomers; shaded histograms: RMSDs of dimers. (A, B) atomistic simulation; (C, D) CG simulation with ELNEDYN network (spring constant: 500 kJ mol^−1^nm^−2^); (E, F) CG simulation with ELNEDYN network (spring constant: 200 kJ mol^−1^nm^−2^); (G, H) CG simulation with IDEN elastic network.

**Figure 7:**
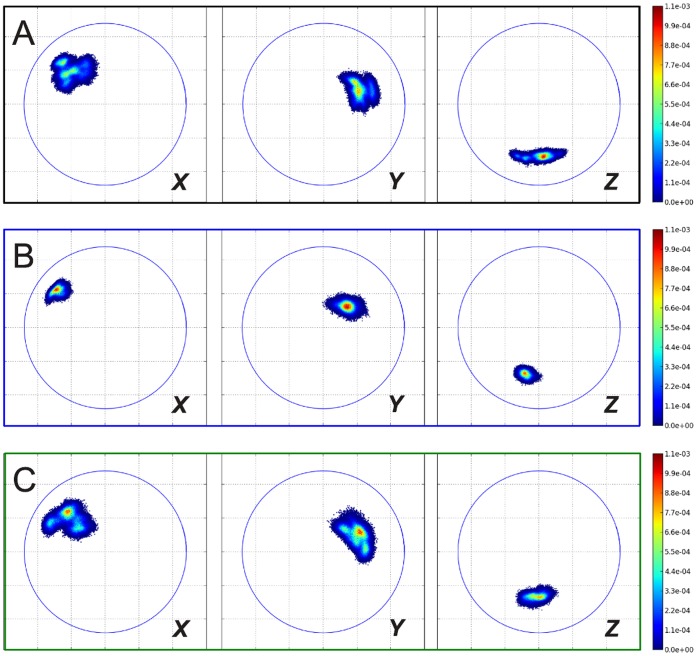
ROMs of inner dimers in simulation of POD+CC complex (see left side of **Figure 6**). (A) atomistic simulation; (B) CG simulation with ELNEDYN network (spring constant: 200 kJ mol^−1^nm^−2^); (C) CG simulation with IDEN elastic network. Projections of the ***X*** *(left panels),* ***Y*** *(middle panels),* ***Z*** *(right panels)*-axis of capsomer 2 on the *xy* plane when capsomer 1 is aligned to the *z* axis (see Methods section). Coloring according to the normalized probability of finding these relative orientations. The blue circles are drawn with a radius of the longest internal axis of the capsomer.

**Figure 8:**
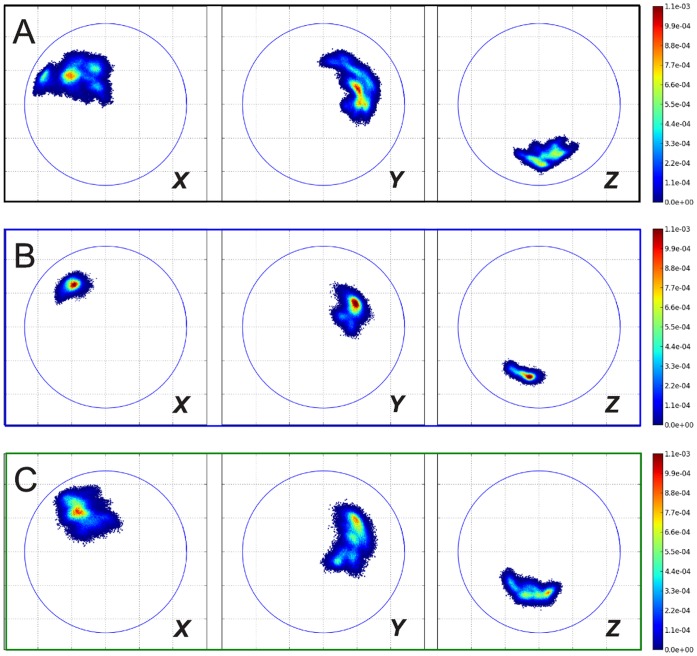
ROMs of outer dimers in simulation of POD+CC complex (see right side of **Figure 6**). (A) atomistic simulation; (B) CG simulation with ELNEDYN network (spring constant: 200 kJ mol^−1^nm^−2^); (C) CG simulation with IDEN elastic network. Projections of the ***X*** *(left panels),* ***Y*** *(middle panels),* ***Z*** *(right panels)*-axis of capsomer 2 on the *xy* plane when capsomer 1 is aligned to the *z* axis (see Methods section). Coloring according to the normalized probability of finding these relative orientations. The blue circles are drawn with a radius of the longest internal axis of the capsomer.

To test the transferability of the local elastic network to larger aggregates we performed simulations of POD+CC complexes and compared them with atomistic simulations (100 ns) of the same system. We used two ELNEDYN networks with spring constants of 500 and 200 kJ mol^−1^nm^−2^ and the optimized heterogeneous network IDEN on top of the MARTINI model. To assess the different models, we analyze the behavior of all 10 dimers within the POD+CC complex, most importantly the RMSD of C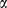 atoms (**Figure 6** and **Table 1**) and the ROMs of dimers (**Figure 7** and **8**).

To ease the analysis procedure we classified the dimers into two groups, inner dimers and outer dimers. The inner dimers are completely “shielded” from all sides by interactions with other capsomers. In contrast, the outer dimers have at least one face which misses interactions that would otherwise be present in a whole viral capsid. We expect these two sets to exhibit qualitatively different ROMs due to the presence of neighbors or the lack thereof. Within each set (inner and outer dimers) the dimers are equivalent by virtue of the 5-fold symmetry of the POD+CC unit; hence we have averaged over the equivalent dimers for the following analyses to obtain better statistics and show results separately for inner and outer dimers.

While the RMSD distribution of C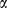-beads in both uniform ELNEDYN networks (spring constant of 500 and 200 kJ mol^−1^nm^−2^, respectively) is narrower than the atomistic reference, the spread obtained with the optimized heterogeneous network IDEN resembles the atomistic simulations (**Figure 6**). With uniform spring constants, the means of the RMSD distributions of individual capsomers (open distribution) are at smaller values compared to the atomistic simulation (**Figure 6C–F** and **Table 1**), i.e. the structural variations within each monomer are too small in these networks. In case of the stiffer uniform network (500 kJ mol^−1^) the backbone fluctuations are essentially restricted to a narrow range centered around the initial conformation. The RMSD distributions of the dimers (shaded distributions) exhibit a similar trend. Naturally, this behavior is more pronounced for the stiffer (500 kJ mol^−1^nm^−2^) network than for the softer one (200 kJ mol^−1^nm^−2^).

The IDEN elastic network in comparison exhibits nearly the same range of RMSD values (both within the monomers and in the dimers) as observed in the atomistic simulation (**Figure 6H** and **Table 1**). The same trend is observed for inner (solid outlines, left panels) and outer dimers (dotted outlines, right panels).

The ROMs of the two sets of dimers (inner and outer) provide a similar picture (**Figure 7** and **8**): while the 500 kJ mol^−1^nm^−2^ uniform elastic network (data not shown to simplify the figure) exhibits a narrower spread, the softer network allows for higher variations for the relative orientations. (A more quantitative comparison of the spreads in the ROM data via means and standard deviations of the orientational distributions is provided in **File S1**, see **Tables S4** and **S5**. These data confirm the qualitative picture discussed above.) Nevertheless, the flexibility gained by homogeneously softening the capsomers does not suffice to reproduce the atomistic behavior of the dimers. Again, the heterogeneous elastic network captures the combination of elasticity of individual proteins and the dimer reorientation dynamics much better.

Note that the structural difference between and AB type dimer and a CC type dimer (RMSD: 0.185 nm, see Supplementary Material) in the crystal structure is much smaller than the typical RMSDs observed within the atomistic simulation of these dimers in the POD+CC complex. This means that the structural variation between different dimeric sites within the CCMV crystal structure is too small to expect a measurable, statistically significant induced fit effect after placing the proteins with the same CG model into two different environments corresponding to an AB or a CC site. However, one does observe that the capsid-like environment poses a restriction to the dimer flexibility that is different for the inner and outer dimers of the POD+CC complex. We find that the optimization of the heterogenous network strength of the IDEN ENM reproduces the subtle restrictive effect of environment on the dimer conformational and internal flexibility and the differences between inner and outer dimers. In contrast, the ELNEDYN networks (with 500 or 200 kJ mol^−1^nm^−2^) too strongly restrict the internal and inter-capsomer motions to observe environmental effects of higher order aggregates.

In the next section we will turn towards whole viral capsids. Here, we will be able to address the question to what extent the elastic behavior of the individual capsomers influences the mechanical properties of the assembled capsid, and, consequently, how a careful parametrization of the elastic network based on atomistic reference simulations of the capsomers improves the ability of the model to actually *predict* said properties.

### Indentation Simulations of CCMV Capsids

In contrast to the POD+CC aggregates described above, studying the mechanics of entire viruses requires system sizes and timescales for which one cannot easily obtain well-equilibrated atomistic reference simulations. We have carried out virus indentation simulations using the CG model(s) and compared them with reference data from AFM (Atomic force microscopy) indentation experiments of full and empty viral capsids of wild type CCMV and mutants [57]. Comparison to these can serve as an independent validation of the proposed optimization route of the IDEN network. The experimentally studied CCMV capsomer closest to that used in our simulations (in terms of primary structure of the capsomer), is the SubE mutant, which carries the point mutation K42R, that causes increased stability compared to the native capsid.

The experiments can be divided into an indentation and a relaxation part. The indentation itself is up to a certain point reversible, but at higher indentation levels hysteresis is observed due to irreversible damage done on the capsid. We compare our results with the experimental ones of Michel et al. [57] from *Fig.3b* (hysteresis observed in SubE) and *Fig.3c* (series of small reversible indentations observed for empty wt-CCMV).

We performed CG simulations with two elastic networks: ELNEDYN with a uniform spring constant of 200 kJ mol^−1^nm^−2^ (this had been for the previous complexes the standard ELNEDYN setup with the best correspondence to the atomistic reference) and IDEN. We performed several simulations with varying speeds of indentation to identify its influence on the force/indentation curves (more details see Methods section). Experiments had been carried out at very slow indentation speeds, in fact so close to reversible/equilibrium condition that no significant influence of the indentation speed on the results had been observed. In the simulation the indentation speed had been kept as slow as feasible (given the enormous computational expense), albeit sill much faster than in experiment. Nevertheless, for the indentation simulations reported in this paper, no dependence of the results on variations of the indentation speed were found, i.e. these simulations were sufficiently close to equilibrium conditions.

**Figure 9:**
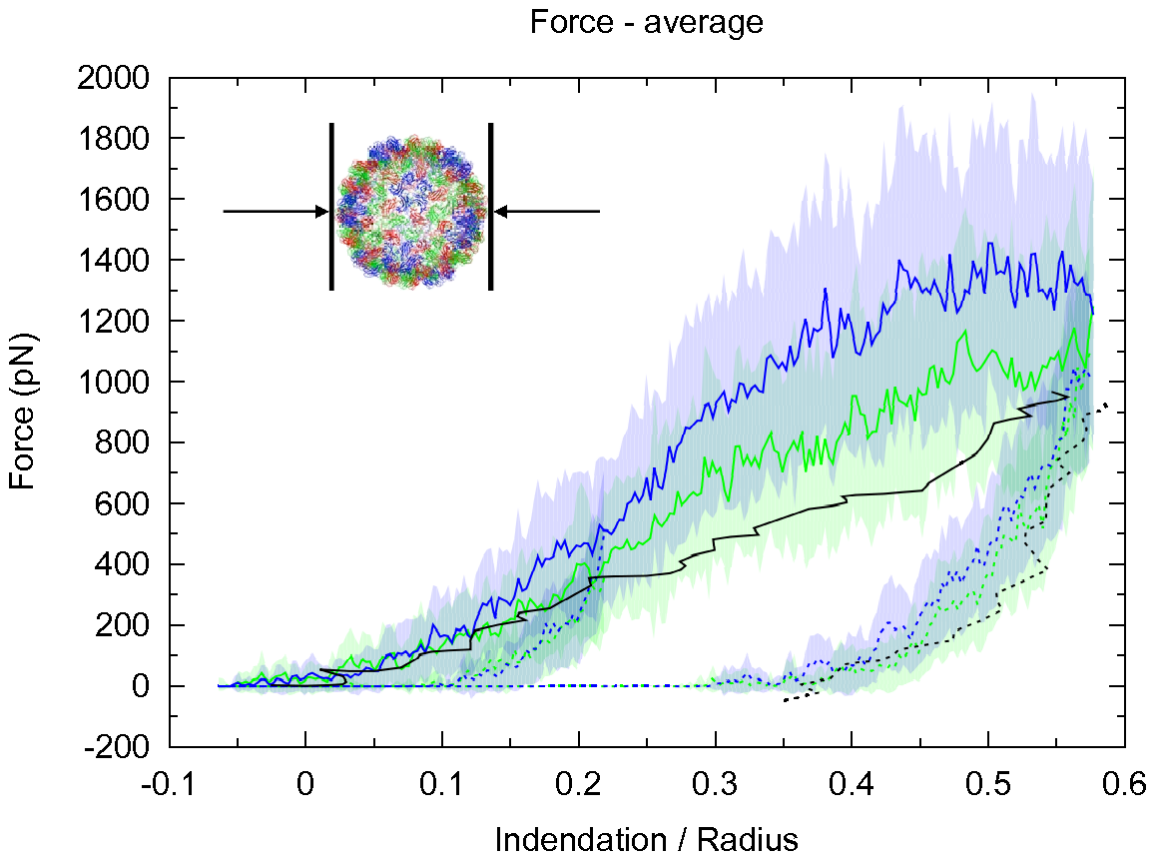
Force distance indentation curves. Comparison of experimental values reported for empty SubE mutants of CCMV [57] (black) and computed values from simulations with an ELNEDYN elastic network with a uniform spring constant of 200 kJ mol^−1^nm^−2^ (blue) and a IDEN elastic network (green). Forces obtained for forward (solid lines) and backward (dashed lines) indentation. The reported forces are average values over the whole 1 ns simulation time of a step and the shaded areas correspond to their respective standard deviation.

**Figure 9** shows the force/indentation curves obtained from both CG models and the corresponding experimental values. Both elastic networks reproduce the experimental data well – with a slightly better agreement for the IDEN model. The simulated virus particles show a largely reversible behavior for small indentations and a stronger hysteresis for larger indentations, with the degree of hysteresis agreeing very well with experimental observations. It should be noted that this irreversible deformation could not be predicted using continuum models as reported in [57].

A previous study by Arkhipov et.al. reproduced the irreversible deformation of a viral capsid in experiments by performing shape based coarse graining (SBCG). The SBCG method effectively reduced each monomer to 15 beads [58], thus eliminating the possibility of tracking back the indentation induced changes in the capsid to the residue level interactions. At the other end of the scale, Zink et.al. performed high resolution (all-atom) indentation simulations of SBMV, albeit at a relatively high indentation speed due to limitations imposed by computational resources [59]. Due to kinetic reasons resulting from the large indentation speeds, this procedure predicted an elastic constant for the capsid that is an order of magnitude larger than experimentally estimated value. These two studies are nicely complementary to the level of resolution employed in the present study which has residue-level interactions while being at the same time coarse enough to perform long simulations.

It is highly encouraging that the model refinement based on *local* (monomer) properties helps to predict *global* properties of the analyzed system. It seems therefore possible to define a valuable elastic network without any free fitting parameters.

## Discussion

In coarse grained protein models, elastic networks are often used as a support to compensate for essential interactions which are lost due to the coarse graining process. We have evaluated how the parameters of these networks influence the properties of CCMV capsomer aggregates and the elastic behavior of entire CCMV capsids.

In the case of the ELNEDYN network we attribute the low flexibility of the capsomer, and consequently of the dimer, to a combined effect of uniform elastic network strength, high value of the elastic network spring constant and “wrongly constrained” regions in the elastic network. When we defined the heterogeneous elastic network we included additional criteria and modifications to the standard ELNEDYN elastic network. Instead of using a simple distance cutoff for defining elastic network bonds and using the initial (crystal) structure as the reference, we defined the network on the basis of *average* distances between atom pairs within the individual proteins obtained from an atomistic MD simulation of a free dimer in solution. We augmented the usual cutoff criterion by two extra conditions: for a bond to be set, the putative bonding partners must either move in a highly correlated fashion or the distance fluctuations between the bonding partner must be small.

Combined with an iterative scheme to match all distance fluctuations with their atomistic counterparts we have arrived at a heterogeneous network that not only captures – by construction – the local elasticity of a capsomer, but also the larger scale fluctuations of capsid fragments, such as the POD+CC complex shown in this paper. Note that while the resulting CG model, i.e. the combination of the MARTINI forcefield and the IDEN elastic network is by construction specific to the protein of interest the approach presented in this paper is easily transferable to other systems. Note also that even though this parameterization demands carrying out atomistic simulations as an input for the iterative procedure, the all-atom resolution simulation is not carried out on the whole complex (viral capsid in our case). Since the CG model should on purpose be based on properties inherent to the individual proteins, only a small subsystem (dimer in our case) needs to be simulated which is relatively “inexpensive”. Moreover, the time scale needed for this reference simulation has to be just long enough to sufficiently sample the (intra) protein fluctuations.

In our atomistic and CG simulations we have observed a wide range of mobility and comparatively large fluctuations in the relative orientations between the two monomers within a dimer. These large fluctuations are greatly diminished when a dimer is in contact with its neighbors. This raises the obvious question: how important is this enormous flexibility of the dimer during the capsid assembly pathway? At this point we can only speculate: for instance, it is an intriguing possibility that this local adaptability is in fact necessary to cover a wide range of dimer configurations that are required during the aggregation process. At any rate, it seems obvious that any computational study aiming to answer this question must (a) reproduce this feature of the fundamental building blocks and (b) be computationally very efficient to cope with the large time scales. We believe that our systematic construction of an efficient CG model with correct elastic properties is an important step into this direction. In addition, the choice of reference system for optimizing the ENM is as important as optimizing the ENM itself. For instance, if we had chosen a larger capsid fragment (such as POD+CC) as a reference for optimizing the IDEN network, that would have resulted in a more rigid ENM which unnaturally limits the capsomers to the conformationally restrictive state within the complex.

We show that local modes of vibration can have a direct and significant effect on protein-protein interactions. Though we have not attempted to simulate the viral assembly process, we did observe that the capsomer flexibility influences and is important in predicting emergent properties like the force response of the capsid under external stress.

In the future, it would be very interesting to see if an IDEN refined elastic network is able to predict structural variations between different units in protein aggregates where induced fit and transitions between different folds upon aggregation plays a significant role.

## Materials and Methods

We base our studies on the crystal structure (2.7 Å resolution) of the salt stable point mutant (K42R) of CCMV (SS-CCMV, PDB code 1ZA7) [60]. In view of keeping things simple (omitting the highly flexible N-terminal tail), we choose the previously mentioned deletion mutant, Δ1–36 (in addition to the point mutation K42R) for this work. In case of chain A, the first 39 atoms are not resolved in the crystal structure of the capsid, so the missing amino acids (residue 37 to 39) were introduced by comparative modeling using MODELLER 9v7 (http://salilab.org/modeller) [61]. Dimeric complexes (starting from the AB dimer structure) and a pentamer of dimers (POD) with adjacent CC-dimers (POD+CC) were studied using atomistic and coarse grained simulations. Finally, we performed indentation simulations of an entire CCMV capsid using existing network models and our newly developed IDEN network, and compared the results with experimental AFM indentation studies [57].

### Atomistic Simulations

The molecular dynamic simulations were performed using the GROMACS simulation package version 4 (4.0.7 for dimer, 4.5.3 and 4.5.4 for higher aggregates) [62]. The system was parameterized using the GROMOS 53a6 force field [63] for protein atoms and the SPC water model [64]. Each protein complex was solvated in a rectangular box and made charge neutral by adding sodium ions. The initial box size was defined such that the minimum distance between the box edges and the protein is 2 nm. All simulations were performed under constant temperature (298 K) and pressure (1 bar). During the equilibration phase the Berendsen weak coupling method was used to maintain the pressure and temperature close to the target values. Coupling constants 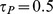 ps for the pressure and 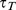 = 0.1 ps for the temperature were applied during equilibration. For the production phase, while the pressure was still maintained using the Berendsen weak coupling method [65], the coupling constant 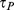 was increased to 5 ps, whereas the temperature was maintained using a Langevin thermostat [66] with a friction coefficient of 1 ps^−1^.

The non-bonded interactions were calculated with a twin-range cutoff scheme. The short range van-der-Waals and electrostatic interactions within a cutoff of 1.0 nm were evaluated every time step, while the long-range van-der-Waals interactions within 1.4 nm were updated together with the neighbor list every 10 time steps. The long-range electrostatics was calculated by the PME method [67], [68] using the default value for Fourier grid spacing of 0.12 nm. In order to allow a time step of 2 fs, all bonds were constrained by the LINCS algorithm [69]. The system was initially energy-minimized with position restraints on the protein atoms (1000 kJ mol^−1^nm^−2^) and subsequently without restraints by steepest descent and later by a conjugate gradient algorithm. Then several 200 ps long equilibration simulations were performed in which restraints were removed in three steps. First, all protein atoms were restrained, then only the backbone atoms, and finally only the C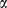 atoms. Finally, several simulations ranging from 100 to 400 ns for the dimeric system were performed. The bigger complex POD+CC, consisting of 20 capsomers, was simulated for 100 ns.

### Coarse-grained Simulations

The CG simulations were performed with the GROMACS simulation package version 4 (4.0.7 or 4.5.4) using the MARTINI force field combined with the ELNEDYN ENM (see below). The atomistic starting structures were coarse grained with the tools provided by the MARTINI developers (http://md.chem.rug.nl/cgmartini).

The MARTINI+ELNEDYN simulations were performed under *NPT* conditions. The temperature (300 K) and pressure (1 bar) were maintained using the Berendsen method with coupling constants 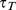 = 0.5 ps and 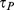 = 1.2 ps, respectively. The non-bonded interactions were treated with a switch function, electrostatic interactions from 0 to 1.2 nm and Lenard-Jones interactions from 0.9 to 1.2 nm. The time step was set to 10 fs and the neighbor list was updated every 5th time step. The box size exceeded the protein outer surface by a minimum of 2.25 nm in each direction. The coarse-grained protein was initially energy-minimized in vacuum for 100 steps of steepest descent. The system was then solvated with water and charge neutralized by addition of sodium ions. The system was again energy-minimized with position restraints applied on all protein beads (1000 kJ mol^−1^nm^−2^). The equilibration step consisted of an initial 50 ps long MD simulation with a time step of 1 fs while applying position restraints to all protein beads. We remind the reader that during setup the term “equilibration” is loosely used to describe the step in the system setup, which is performed to ensure it is in a relaxed state. It by no means suggests *thermal* equilibration. Then a 1 ns long simulation with the same restraints but with increased time step of 10 fs was performed. Subsequently, another 1 ns long MD simulation with restraints applied to the backbone beads was carried out. Finally, all restraints were lifted for executing production simulations.

In any CG simulation an effective speed-up of the dynamics is expected due to the smoothening of the free energy landscape. Therefore, one typically determines the time-scaling factor that relates the accelerated CG dynamics to realistic timescales obtained from experiments or atomistic reference simulations. According to the authors of the MARTINI forcefield and the ELNEDYN ENM, this time-scaling factor is approximately 4 for the MARTINI model (after analyzing the diffusion of CG water and lipids) [55]. In the present paper, we do *not* generally rescale the timescales reported for the CG simulations by this factor of 4, only at some instances (for example to estimate the indentation speed in the CG simulations) we report also these rescaled times (indicated with an asterisk) to allow for an approximate comparison with real world times and experiments.

### Construction of Elastic Networks

In this paper we study two types of elastic network. The first one, ELNEDYN, was proposed by the developers of the MARTINI CG model. The second one, IDEN (Iteratively-refined Distance-based Elastic Network), is a refined version of this, in which both the question of whether or not a bond is placed and its ultimate strength are determined by making use of information from an underlying atomistic trajectory. Let us now describe the two network models.

#### ELNEDYN

The ELNEDYN network [38] is defined as an anisotropic elastic network [40], [43]. ELNEDYN is also homogeneous, having a uniform bond strength assignment for all elastic network bonds. Any two C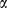 beads *i* and *j* are connected by a harmonic bond of spring constant *K* and rest length 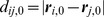 (as measured in the crystal structure) when two criteria are met: first, they are at least 2 steps apart along the backbone, and second, 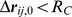, for some cutoff distance 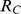. The value of both parameters is chosen such as to reproduce the RMSF values of atomistic simulations with the CG model, as described in Ref. [38] (see **Figure S4 and S5** in **File S1**).

#### IDEN

We wish to go beyond the standard ELNEDYN construction in two ways. First, we propose a more refined criterion for when two C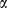 beads are connected by a spring; and second, rather than using a single spring constant 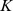 for every harmonic bond, we implement an iterative scheme to optimize the spring constants individually, thus setting up a locally heterogeneous network. This possibility was first explored by Lyman et al. [48], but these authors did not consider an additional CG force field next to their elastic network.

The usual criterion for establishing a bond once two C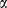 beads are closer than some cutoff can lead to erroneous bonds, because these beads might just be accidentally close in the crystal structure (or an ensemble average), but in fact show large dynamic excursions away from each other. For instance, part of a flexible tail of a protein might be close to the core in the available structure, but it indeed explores a wide phase space away from that conformation and permanently linking it to the protein core would un-physically restrict its motion. Hence, we need a criterion to judge whether two neighboring beads should be linked that goes beyond their mere distance. We propose that their positional correlations and the variance of their distance, both measured for instance through an MD trajectory, will help to address this issue.

For a list of atom coordinates 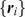 taken from a trajectory (or indeed any other ensemble we wish to average over) the (scalar) covariance of an atom pair *i* and *j* is given by
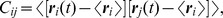
where 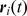 and 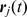 are the coordinates of atoms *i* and *j* at time *t* and 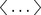 denotes an average over the trajectory (or ensemble). To eliminate overall factors, we further define the correlation coefficient, 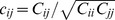 which is bounded between −1 and 1 (by virtue of the Cauchy-Schwarz inequality). In the crystallographic literature this is often referred to as the “per atom normalized covariance” (PANC) [70], but we see no need to introduce a new name here.

To define and calculate 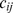, we must first fit all protein frames to a reference (e.g. initial) frame, in order to remove overall translations and rotations. Unfortunately, this also removes any significant correlation between the fairly rigid core atoms and would erroneously suggest that they are uncorrelated. We resolve this issue by additionally monitoring distance fluctuations between atoms as a secondary criterion for bonding. Hence, define the (scalar) distance 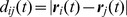 between two atoms *i* and *j* and its variance
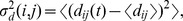
which can be computed without the fitting procedure necessary to obtain 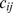. Our procedure to identify the network is now as follows: A harmonic bond will be established between all pairs of C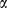 beads *i* and *j* which satisfy the following criteria:

1. They are at least 2 residues apart along the backbone sequence.
2. Their average distance 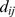 is less than a pre-specified cutoff distance 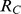. We follow the ELNEDYN choice of setting 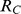 = 0.9 nm.
3. In addition:
  Their correlation coefficient is sufficiently high; we used 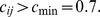

  OR
  The variance of their distance is smaller than a maximum; we used 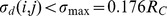.

The criterion for selecting an 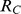 cutoff value of 0.9 nm follows the recommendation by the authors of MARTINI and ELNEDYN elastic network [34], [35]. They tested various cutoff values and established 0.9 nm and an elastic network bond strength of 500 kJ mol^−1^ nm^−2^ to be the best compromise between the network density and its stiffness. We tested various values for the correlation coefficient and the distance fluctuation criteria. Additional information regarding the choice of these parameters can be found in **File S1** (see **Figures S6** and **S7**).

After having identified the bonds, we now determine the spring constants. We first scale the initial value 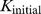 = 500 kJ mol^−1^nm^−2^, as typically used for ELNEDYN networks [38], according to the following procedure:
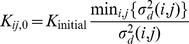



This implies that the strongest bond we place will have spring constant 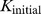, and that for every specific bond this value is scaled down inversely proportional to the fluctuations of that bond. The latter were taken from a 400 ns atomistic reference trajectory of a dimer. Since we aimed to create one universal elastic network for all capsomers, the two chains (A and B) from the atomistic dimer simulation were separately extracted and averaged for the analysis. In other words, a 400 ns long atomistic simulation of the dimer converts to 800 ns of statistics for a capsomer.

In the next step we iterated the strength of all spring constants 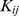 in order to reproduce the fluctuations of the atomistic reference simulation. In contrast to a pure elastic network construction this cannot be done analytically (say, by some matrix inversion), because the (non-harmonic!) CG force field also contributes to the distance fluctuations. We investigated two different update rules: The first one is inspired by the one proposed by Lyman et al. [48]:
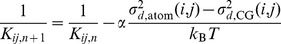



This suggests itself because (i) dimensionally the spring constant should be inversely proportional to the variance and (ii) the latter proportional to the thermal energy. We used the value 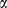 = 0.05. We also tried a direct iteration according to
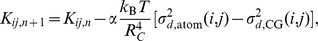
where we used 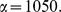


For the studied CCMV system we observed that the convergence was faster with the direct scaling method. We declared convergence for our refinement procedure once the difference between atomistic and coarse-grained simulation in the distance variances 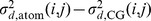 averaged over all pairs (i,j) is converged (for details see the corresponding section in **File S1** including **Figure S8, S9** and **Table S3**). This was typically the case after 11 iteration steps, further iterations did not improve the agreement.

We emphasize once more that we introduce a supporting elastic network only within single capsomers, not between them (i.e., we do not link dimers or higher order aggregates). One may suspect, and our simulations supported this, that the much slower inter-capsomer fluctuations would necessitate bonds that are extremely weak and not necessarily harmonic. If we wish to understand genuinely nonlinear responses, such as the breaking of a capsid upon major compression, it is impermissible to string the entire capsid together and thereby eliminate putative failure modes for irreversible damage.

### Coarse Grained Indentation Simulations

The setup of the whole capsid followed the general procedure described above and used two different kinds of elastic network (ELNEDYN and IDEN) as described. Unlike the procedure described above, for the indentation simulations the last step in the equilibration scheme was extended from 1 to 10 ns in order to allow for a solvent exchange between the inside and outside of the virus. We performed these indentation simulations to compare with experiments performed on CCMV by Michel et al. [57]. Our indentation simulations were repeated for indentation speeds of 0.04–0.02 nm ns^−1^ corresponding to 0.01–0.005 nm ns^−1^* in “real time”. These checks were necessary to verify that these large indentation speeds nevertheless had no major effect on the force/indentation curves, recalling that the indentation speeds reported for experiments are much slower, between 20–2000 nm s^−1^. On the other hand, experiments do not report any influence of varying the speed by two orders of magnitude on the force/indentation curves [57].

#### Forward indentation

We performed indentation by adapting a method developed for the calculation of osmotic pressure [71]. In this method, two semi-permeable repulsive walls (10-4 Lennard Jones potential interacting only with the protein beads and not with the solvent molecules or counter ions) are used to laterally constrict the capsid. Initially, the walls are placed just beyond the viral capsid surface, but behind the interaction cutoff of the wall potential. The indentation process was simulated by subsequent stepwise shrinking of the distance between the walls from 29 nm to 20 nm in 0.04 nm steps and simulating for 1 ns for each step. To replicate the experimental setup of Michel et al. [57], two indentation setups, one starting from a wall distance of 29 nm to 25 nm and the other starting from 29 nm to 20 nm were devised. The difference being, the former indentation is “*reversible*” and the later “*irreversible*”. An indentation is “*reversible*” if the force versus indentation curve of the viral capsid under external stress returns to the starting state, when the external stress is released. These indentation setups were simulated on IDEN and ELNEDYN networks.

#### Relaxation

Relaxation of the *“fully squeezed”* viral capsid was performed by slowly expanding the distance between the walls in 0.04 nm steps and simulated for 1 ns at each step. Two relaxation simulations with the initial state starting from *reversibly* and *irreversibly squeezed* capsid were performed, for both the CG MARTINI forcefield combined with ELNEDYN and IDEN elastic networks. The simulations were terminated when the separation between the semi permeable walls was 29 nm. Reported forces are either running averages over 10 or 50 values (10 ps steps) from simulation or an average over the whole 1 ns simulation time.

### Analysis: Relative Orientation Maps

In order to distinguish between the relatively rigid core of the capsomer and the flexible N-terminal and C-terminal tails, a core region spanning from residue 14 to 142 was defined omitting the flexible tails (see **Figure 1D**). Within each capsomer, an internal reference frame was defined to facilitate the calculation of relative spatial orientations between capsomers in aggregates. **Figure 1D** visualizes the capsomers with our choice of internal axes, which is the following: The center of mass of the capsomer was chosen as the origin of the internal frame of reference. The ***X, Y*** and ***Z*** axis of the internal frame were defined by guides connecting the origin and the centers of mass of clusters of C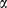-atoms within the protein (***X***-axis: C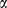-atoms of residues 69–71, 92, 122–124; ***Y***-axis: C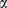-atoms of residues 19–21,136; ***Z***-axis: C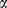 of residues 55–57, 137–139). These atoms were chosen such that the resulting axes were approximately orthogonal to each other. Using C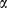-atoms to define the three internal axes has an inherent disadvantage of loss of exact orthogonality between the axis due to atom fluctuations during the course of the simulation. This was done since using the eigenvectors of the gyration tensor instead is difficult, because the two smaller eigenvalues are so close that their corresponding eigenvectors easily swap identity or rotate uncontrollably around the axis defined by the largest eigenvalue. We verified that this has only a negligible effect on the observables in our analysis (data not shown).

**Figure 10:**
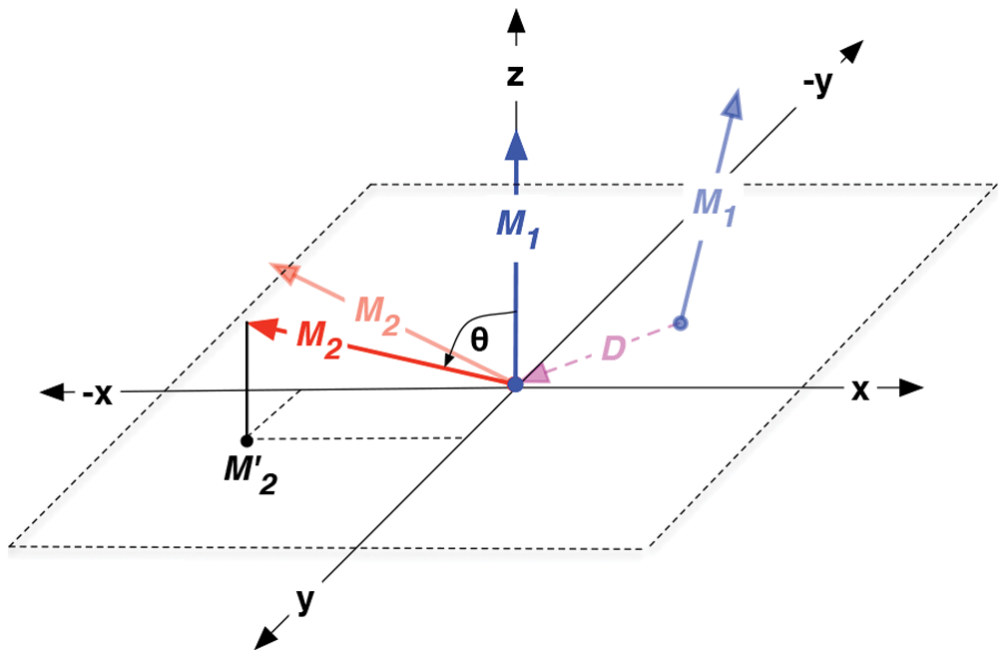
Illustration of the procedure to analyze the relative orientation between the capsomers within a dimer as described in the main text. 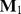
 and 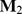 are the corresponding axes (*X*, *Y* or *Z*) of each capsomer in the dimer. D is the distance between the center of mass of the capsomers and 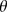 is the angle between the axis 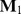 and 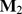 and the lightly shaded vectors indicate the original axis orientations in the dimer before aligning and rotating the internal frames.

Since the dimer is the smallest stable structural component and forms the basis of the CCMV capsid assembly, we chose dimers as the basis of our analysis. **Figure 10** illustrates the procedure: Vectors 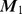 and ***M***_2_ are two corresponding axes of the internal frame (***M*** takes the value of either ***X, Y*** or ***Z***) for capsomer 1 and capsomer 2, respectively, for each dimer. Non-capitalized *x, y* and *z*-axes refer to the global external frame of reference. *D* is the distance between the center of mass of the capsomers and 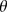 is the angle between the axes ***M***_1_ and ***M***_2_ and *t* is the current time step of the data being plotted. The analysis procedure is outlined below.

1. One of the two capsomers in a dimer (capsomer A in the case of an AB dimer and an arbitrarily chosen one in the case of a CC dimer) is denoted as “capsomer 1” and is fit to the starting structure of the AB dimer (using rigid core atoms) from the 400 ns reference simulation, thus eliminating rotational and translational motions of the dimer system in the trajectory. It should be noted that all coarse-grained and atomistic simulations were fitted to the same starting structure.
2. For every frame of this fitted trajectory, each pair of internal axes is rotated as a rigid body such that the ***M***_1_-axis aligns with the global 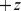 direction (in case of 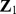 and 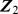 in the 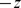 direction, for better visualization). As elaborated above, all arbitrary rotations about the 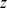-axis are eliminated because of fitting to the same reference structure.
3. The projection 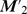 of 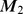 on the *xy*-plane is calculated.
4. The points in the scatter plot of projections are colored according to the probability density.

## Supporting Information

File S1 **Supplementary material regarding the structure of the capsomers and dimers on the different symmetry inequivalent sites of the CCMV capsid is shown.** In addition, further technical details of the setup and parameters of the IDEN elastic network and the convergence of the iterative optimization of the strength of the IDEN elastic bonds are presented. Furthermore, to better characterize the relative orientations between the two capsomers in the atomistic and CG simulations of the free dimers and the dimers in the POD+CC complex, the spread in the relative orientation maps (ROMs) is analyzed quantitatively.
(PDF)
