# Supplementary material for: Optimization of an Elastic Network Augmented Coarse Grained Model to Study CCMV Capsid Deformation
Source: PLoS One. 2013 Apr 16;8(4):e60582. doi: 10.1371/journal.pone.0060582 (PMC3628857; doi:10.1371/journal.pone.0060582)
Supplement: File S1 — Supplementary material regarding the structure of the capsomers and dimers on the different symmetry inequivalent sites of the CCMV capsid is shown. In addition, further technical details of the setup and parameters of the IDEN elastic network and the convergence of the iterative optimization of the strength of the IDEN elastic bonds are presented. Furthermore, to better characterize the relative orientations between the two capsomers in the atomistic and CG simulations of the free dimers and the dimers in the POD+CC complex, the spread in the relative orientation maps (ROMs) is analyzed quantitatively. (PDF) [file pone.0060582.s001.pdf]

Supplementary material to:

Optimization of an Elastic Network Augmented Coarse Grained Model to Study CCMV Capsid Deformation

C. Globisch, V. Krishnamani, M. Deserno, C. Peter

Comparison of site-inequivalent subunits of the CCMV capsid

The CCMV capsid is constructed from 180 copies of the same protein. Since in the $T = 3$ capsid geometry three symmetrically inequivalent locations for these proteins exist, their *folds* in these three sites – labeled A, B, and C – are slightly different. Their RMSD deviations (for the entire protein or only its central core) are compiled in Tab. S1; the deviations are visualized in an overlay of the three x-ray structures in Fig. S1 (taken from Speir *et al.*, J. Virol. **80**(7), 3582-3591 (2006)). The biggest differences for the wild type monomer occur at the N-terminal tails, but in our study we use the Δ1-36 mutant where these parts are mostly clipped (the remaining parts at the N-terminal tail are found in the lower left corner in Fig. S1). For these mutants the most relevant differences occur in the C-terminal tails (lower right corner in Fig. S1), which determine also the interface between and the relative orientation of the monomers in a dimer complex. Tab. S2 and Fig. S2 repeat this analysis for dimers, i.e. provide a comparison between the two inequivalent types of dimers (AB and CC type) that occur in the CCMV capsid.

Table S1: **RMSD (nm) between A, B and C-type monomers.**

C_α atoms (nm) – initial atomistic structures (Δ1-36)		
Monomers	C_α	C_α core (residues 50-178)
A vs. B	0.200	0.035
A vs. C	0.203	0.032
B vs. C	0.062	0.020

Table S2: **RMSD (nm) between AB and CC type dimers.**

C_α atoms (nm) – initial atomistic structures (Δ1-36)		
Dimers	C_α	C_α core (residues 50-178)
AB vs. CC	0.213	0.185

Let us add one more comment on the N-terminal structural differences: These manifest at the five-fold and three-fold symmetry sites where proteins come together: While three B- and three C-monomers join their N-terminal tails into a β-barrel at the hexameric (= three-fold symmetry) center, there is no predominant structural motif at the pentameric symmetry site where five A-type monomers meet (for a recent computational study see Bereau *et al.*, J. Chem. Theory. Comput. **8**(10), 3750–3758 (2012)). The differences in the folds would start to appear at the cleavage site of the mutant, so constraining the tails there by an elastic network to a structure of the B- or C- type monomer would prevent a transition to the A-type monomer.

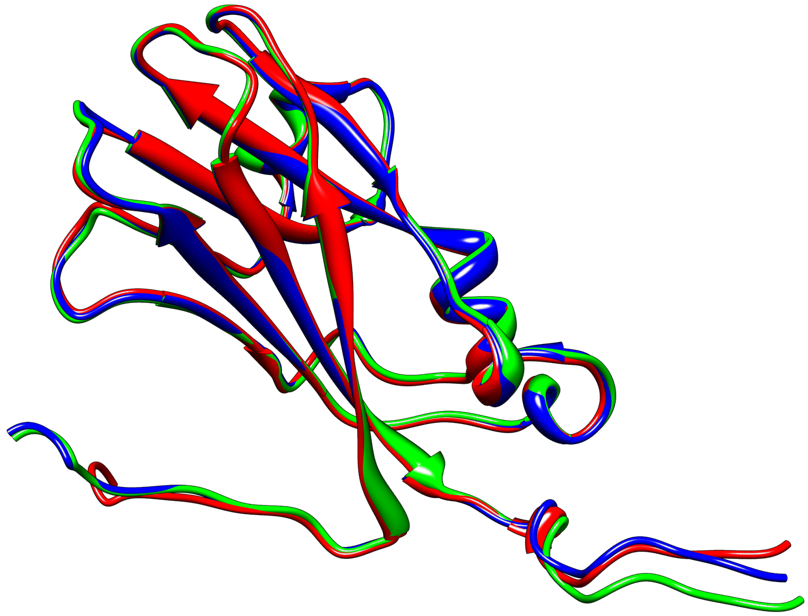

Figure S1: **Aligned monomers of CCMV (Δ1-36).** Chain A (blue), chain B (red), chain C (green).

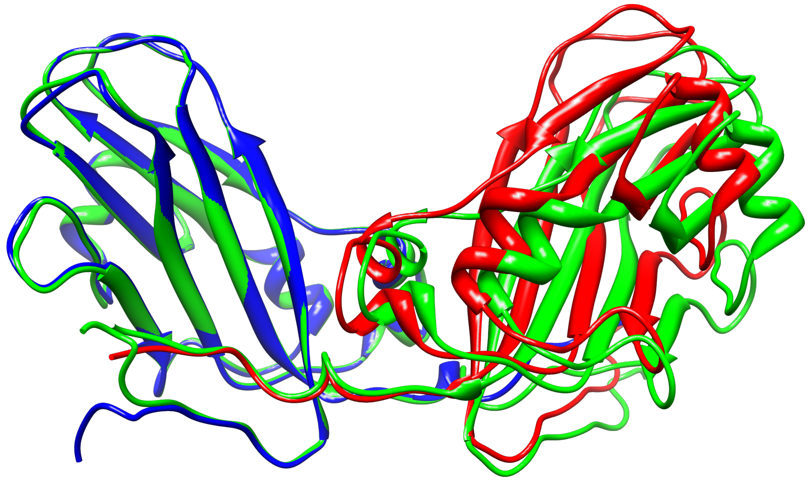

Figure S2: **Aligned dimers of CCMV (Δ1-36).** AB type dimer (red/blue), CC type dimer (green). The alignment is enforced on the left side, by optimizing the superposition of an A-type and a C-type monomer.

Twist angle of the dimers

Fig. 2C and D of the main text considers a "twist angle" between the two monomers of a dimer. Fig. S3 illustrates its definition. A plane is defined by selecting the center of mass of 3 clusters within the rigid core of each monomer. The clusters are defined as the center of mass of the C_α atoms of following residues: Cluster 1 = 41-43, 46-49, 111-113; Cluster 2: 25-30, 124-130; Cluster 3: 80-87 (Note: the residue numbers are given with respect to the simulated deletion mutant starting with residue 37, therefore residue 41 in our simulated structure corresponds to residue 87 in the wild type structure). The angle between the two orthogonal plane vectors defines the twist angle.

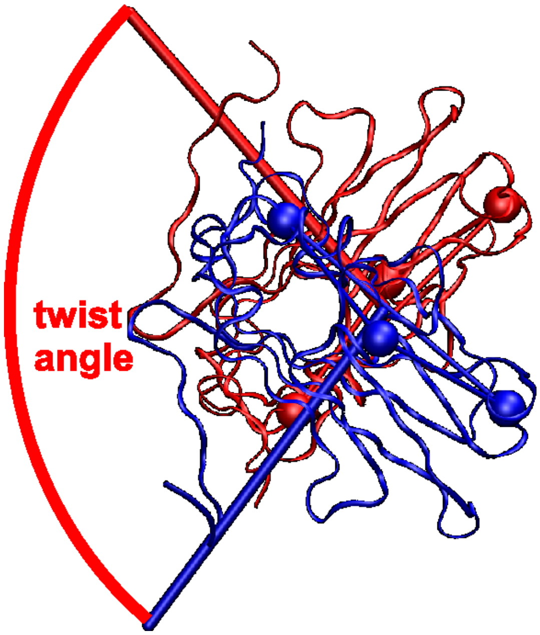

Figure S3: **Twist angle.** The two monomers are colored blue (type A) and red (type B).

Comparison of RMSF values for AB dimer simulations

The construction of a network influences not only the stiffness of individual monomers, but also those of the dimers—especially since the large scale motions at the hinges are strongly affected. Fig. S4 illustrates the extent of these fluctuations in three independent *atomistic* simulations of an AB dimer: one 400 ns long (black) and two 100 ns long (red and magenta); notice that the results are compatible with each other, meaning we have indeed reliable access to the RMSF values. We subsequently used the 400 ns trajectory as our reference. Figure S5 compares RMSF values for an *AB dimer*, obtained in an atomistic reference trajectory, with values obtained from coarse grained simulations, using the ELNEDYN parameters K500 and K200. The K500 parameters clearly make the dimer too stiff, but even the K200 parameters miss several important flexible regions. As the Fig. S9 (right) in the following section will show, the iterated IDEN network does a much better job in rescuing the magnitude of these RMSF values.

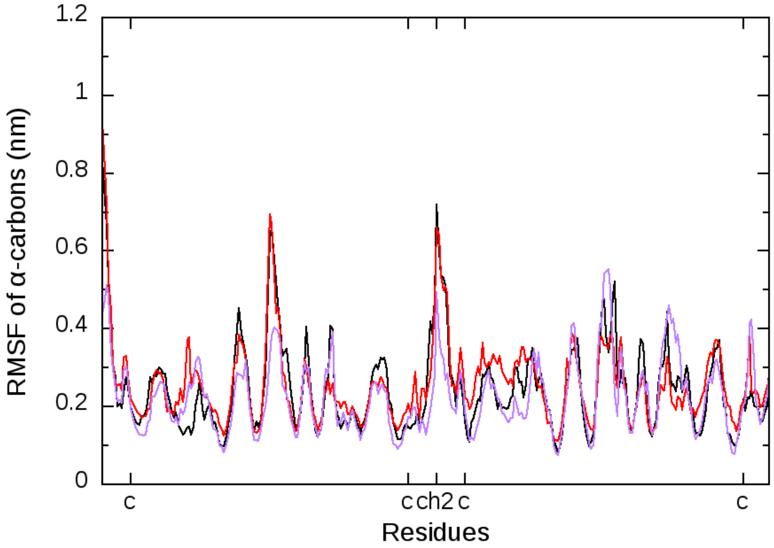

Figure S4: **RMSF values in three independent atomistic simulations of an AB dimer.** The figure shows the 400 ns long reference simulation that was used for the iteration procedure (black), as well as two independent 100 ns long simulations (red and magenta).

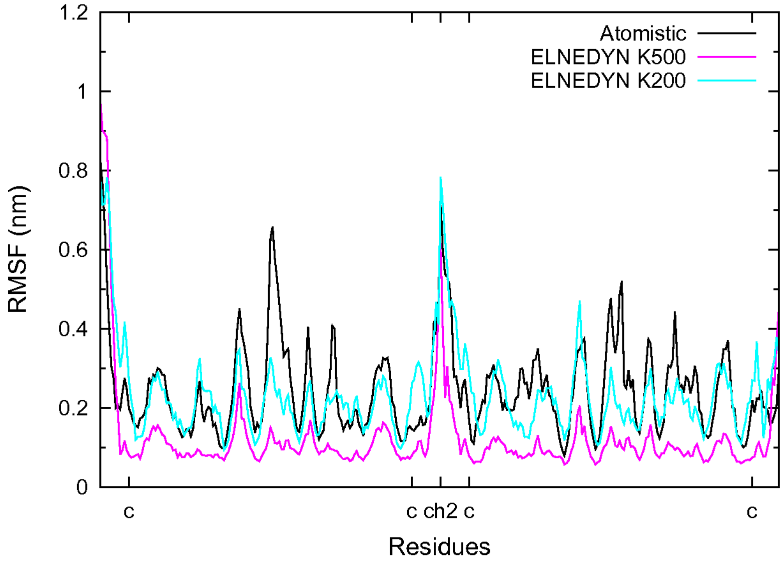

Figure S5: **RMSF values of ELNEDYN simulations compared with the atomistic reference.**

Variance and correlation cutoff for the IDEN elastic network

Figure S6 shows the variance of C_α distances in the atomistic reference simulation between all C_α pairs for which the standard ELNEDYN criterion would result in the creation of a network bond (pairs are within 0.9 nm and separated by at least 2 residues along the sequence). Observe that some of these distances

fluctuate quite substantially in the atomistic simulation, so it seems unwise to permanently link their corresponding C_α atoms. Our variance cutoff choice of 0.025 nm^2, which corresponds to a standard deviation of about 1.6 Å (or 0.176 R_C), ensures that no bonds are placed between too strongly fluctuating atom pairs.

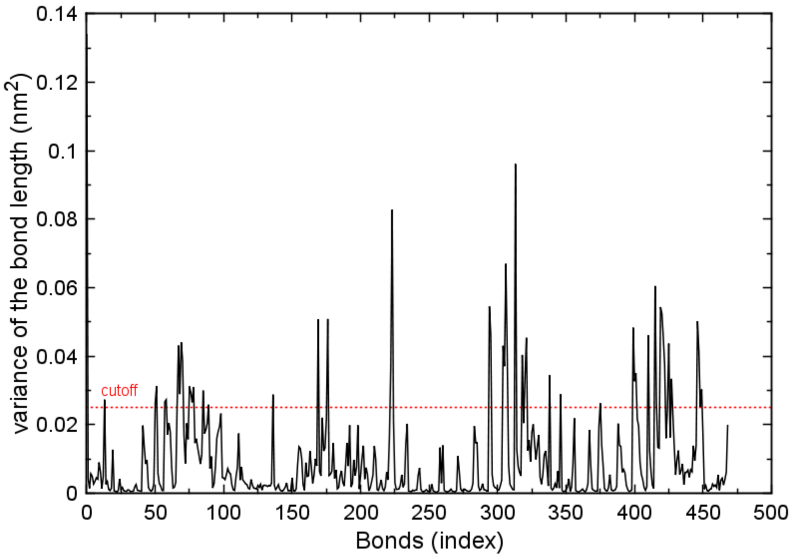

Figure S6: **Variance of C_α bond lengths extracted from atomistic reference.** Choosing a cutoff at 0.025 nm^2, such that no bonds are set between C_α pairs with a bigger variance, ensures that pairs with strongly fluctuating distances are not linked by a bond.

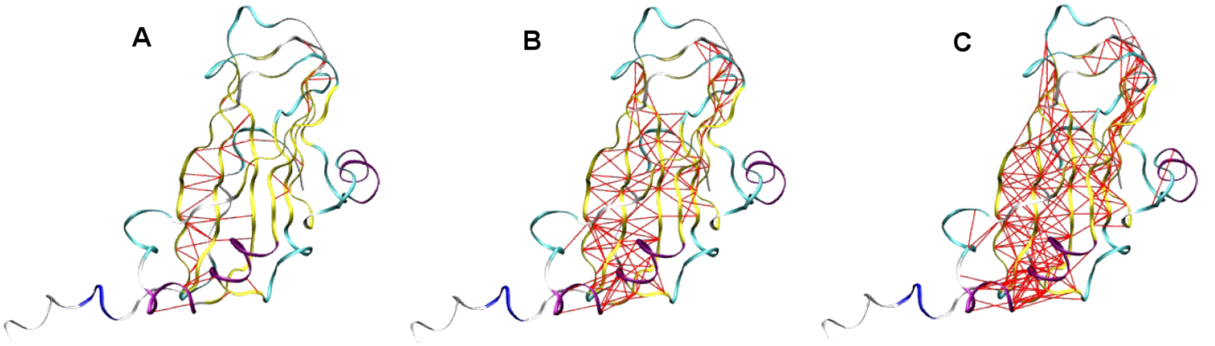

Figure S7: **Influence of the correlation cutoff $c_{ij} > c_{\mathrm{min}}$ on the density of the elastic network.** All C_α pairs within 0.9 nm and separated by at least 2 residues in the sequence are considered; c_{min} takes the values 0.8, 0.7, and 0.6 in panels (A), (B), and (C), respectively.

In addition to the *variance of distances*, we also use the *correlation coefficient* c_{ij} between the positions of two C_α atoms, after mutual alignment. Figure S7 shows how different choices for the correlation cutoff influence the bond definition. Generally, a high correlation cutoff (e.g. $c_{\mathrm{min}} = 0.8$, see Fig. S7A) is restrictive and leads to only few C_α pairs being bonded because of correlated motions, while a low correlation cutoff (e.g. $c_{\mathrm{min}} = 0.6$, see Fig. S7C) is generous and results in many more bonds. Note that the criterion of

low distance variances already introduces beads in rigid regions with low distance fluctuations. Thus the covariance criterion is mostly important for regions with higher mobility. Here one wants to have pairs bonded that move in a comparatively correlated fashion. We selected $c_{\min} = 0.7$ as a compromise. Notice, though, that the physics of the situation permits a range of values without too much affecting the result: As we permit more and more bonds (by reducing $c_{\min}$), their correlation drops and presumably their distance fluctuations rises, implying that the bonds we end up placing would after the iteration procedure be not particularly strong.

Convergence criterion for the iterative optimization of IDEN

During iterative optimization of the network we monitor the difference between the bond fluctuations $\sigma_d^2(i,j)$ in the atomistic reference and the bond fluctuations $\sigma_{d,\mathrm{CG},n}^2(i,j)$ in the coarse grained simulation (at iteration n). The difference $D_n(i,j) = \sigma_d^2(i,j) - \sigma_{d,\mathrm{CG},n}^2(i,j)$ is thus a variable of the bond pairs (i,j) and during the iteration process we average over all bond pairs and monitor $\langle D_n \rangle$. In Fig. S8 we plot $\langle D_n \rangle$ and its standard deviation σ_{D_n} as a function of iteration number n, normalized by the value at $n = 0$ (i.e., the original value before we have iterated). The absolute numbers can be found in Tab. S3. We see that both $\langle D_n \rangle$ and σ_{D_n} drop from their initial values and approach equilibria beyond which further iterations do not substantially improve the result anymore. Based on such plots one can decide when one stops the iterative refinement. We decided to use the values obtained at step $n = 11$ for our subsequent analysis; both mean and deviation appear to have converged, and the mean is actually very low.

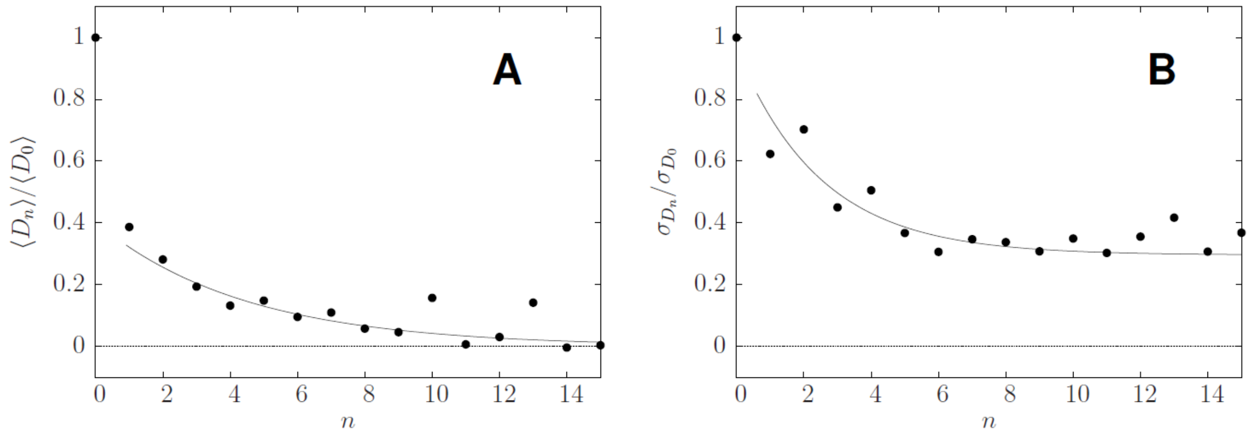

Figure S8: **Iteration steps with direct scaling.** Defining the random variable $D_n = \sigma_d^2(i,j) - \sigma_{d,\mathrm{CG},n}^2(i,j)$ as a measure for the difference between all-atom and coarse grained network fluctuations, we can monitor its (scaled) mean $\langle D_n \rangle / \langle D_0 \rangle$ and (scaled) standard deviation $\sigma_{D_n} / \sigma_{D_0}$ as a function of iteration number n in order to monitor convergence.

As the iteration proceeds, both the variance between bond lengths and the RMSF values of residues, as measured in the coarse grained simulation, approach the values from the atomistic reference trajectory that we intend to reproduce. Fig. S9 illustrates that this works very well by comparing the atomistic reference with the CG data before iteration and the CG data after 11 iterations.

Table S3: **Iteration steps with direct scaling.** Mean $\langle D_n \rangle$ and standard deviation σ_{D_n} of the difference variable $D_n = \sigma_d^2(i,j) - \sigma_{d,\mathrm{CG},n}^2(i,j)$, as obtained over the ensemble of all bonds (i,j). Notice that $n = 0$ corresponds to the starting situation before any iterations have been performed.

Deviation to aa ref.	Initial	Iter 1	Iter 2	Iter 3	Iter 4	Iter 5	Iter 6	Iter 7
$\langle D_n \rangle$	0.01058	0.00409	0.00298	0.00205	0.00140	0.00157	0.00101	0.00116
σ_{D_n}	0.01173	0.00731	0.00824	0.00528	0.00593	0.00431	0.00359	0.00407

Deviation to aa ref.	Iter 8	Iter 9	Iter 10	Iter 11	Iter 12	Iter 13	Iter 14	Iter 15
$\langle D_n \rangle$	0.00061	0.00049	0.00166	0.00007	0.00032	0.00150	-0.00004	0.00004
σ_{D_n}	0.00396	0.00361	0.00410	0.00355	0.00417	0.00489	0.00360	0.00432

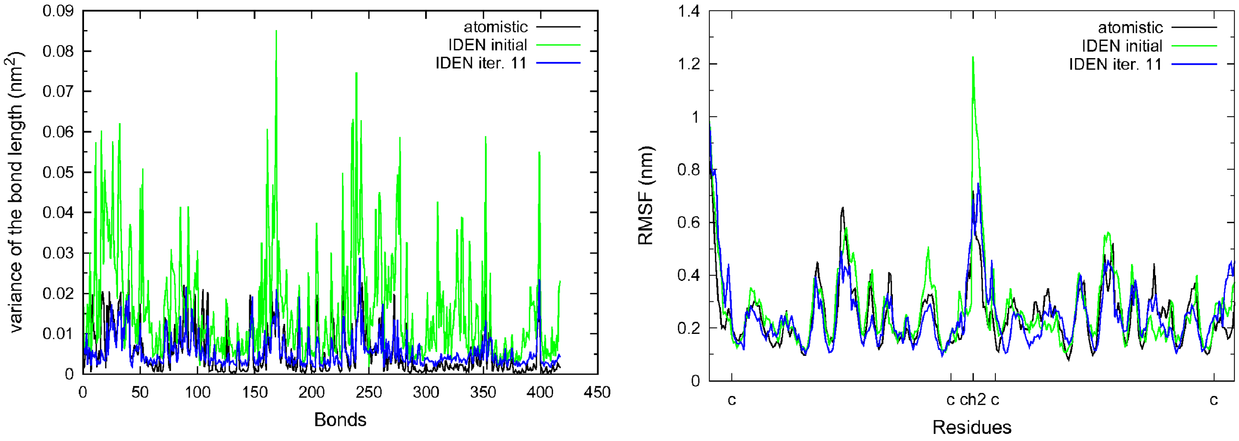

Figure S9: **Converging fluctuations.** Variance of bond lengths for all bonds (left) and RMSF values at all residues (right) for the atomistic reference trajectory (black), initial CG network (green) and final IDEN CG network after 11 iterations (blue).

Additional analysis of the ROMs

The relative orientation maps (ROMs) in the main text are a way to visualize the relative motion of a dimer, by monitoring the projections of some chosen dimer axis onto the orthogonal plane of another chosen axis of the second dimer. Unfortunately, the three-dimensional geometry is very hard to conceptualize in a concrete way, but it turns out that this is not particularly important, as long as one can ensure that the orientational spread of all independent projections is comparable between atomistic target and coarse grained simulation.

Should one wish to further quantify the ROMs, then there are several possibilities. One is to consider the ROM distribution functions in polar coordinates and *marginalize* them into *radial* and *angular* distributions, and for the latter one can easily determine mean and standard deviation. This is done in Tab. S4 for the X- and the Z-projection. However, the same procedure does not work well for the Y-projection, because it is centered, and as such an angular spread does not make sense. An alternative approach (which abandons any approximate angular symmetry between the two sets of angles but works under all conditions) is to marginalize over the *cartesian* (horizontal and vertical) projections of the ROM, as illustrated in Fig. S10. Tab. S5 summarizes these latter marginalizations for dimers in the larger POD+CC simulation, corresponding to Figs. 7 and 8 of the main manuscript. In that case, the five AB dimers are central (lying in the "inner circle"), while the five CC dimers are peripheral (lying in the "outer circle").

Table S4: **Statistics of ROM distributions of capsid protein dimer and capsomer (monomer).** Data obtained from isolated dimer (Figure 4 in the main text) and POD+CC simulations (Figure 7 and 8 in the main text), using Atomistic, ELNEDYN with a uniform elastic network constant of 500 or $200\,\mathrm{kJ\,mol^{-1}nm^{-2}}$ or refined IDEN elastic network. The radial and angular distributions are obtained as polar coordinates with respect to origin $(0, 0)$.

	X component		Z component	
	Radial (nm)	*Angular (deg)*	*Radial (nm)*	*Angular (deg)*
**Atomistic**				
Dimer	8.497 ± 0.647	138.130 ± 9.868	7.054 ± 0.576	221.195 ± 14.158
POD+CC Inner Dimer	7.210 ± 1.133	135.097 ± 10.752	7.816 ± 0.380	271.420 ± 10.709
POD+CC Outer Dimer	6.477 ± 1.948	131.517 ± 18.650	7.917 ± 0.736	278.534 ± 12.311
**ELNEDYN** ($K500$)				
Dimer	8.852 ± 0.492	150.877 ± 5.916	4.154 ± 1.265	244.890 ± 8.957
POD+CC Inner Dimer	8.372 ± 0.437	141.737 ± 5.028	7.388 ± 0.272	253.159 ± 5.949
POD+CC Outer Dimer	7.667 ± 0.721	131.837 ± 6.280	7.438 ± 0.343	251.306 ± 7.842
**ELNEDYN** ($K200$)				
Dimer	7.433 ± 0.754	165.838 ± 7.210	6.917 ± 0.391	227.780 ± 6.098
POD+CC Inner Dimer	8.947 ± 0.506	142.022 ± 4.078	7.164 ± 0.395	254.115 ± 4.441
POD+CC Outer Dimer	7.958 ± 0.549	127.975 ± 5.761	7.421 ± 0.335	253.793 ± 8.697
**IDEN Network**				
Dimer	5.104 ± 1.326	170.929 ± 10.910	5.450 ± 0.717	234.491 ± 9.180
POD+CC Inner Dimer	7.380 ± 1.438	137.181 ± 9.036	6.707 ± 0.441	266.890 ± 8.617
POD+CC Outer Dimer	7.098 ± 1.579	120.575 ± 11.794	6.296 ± 0.598	260.047 ± 18.480

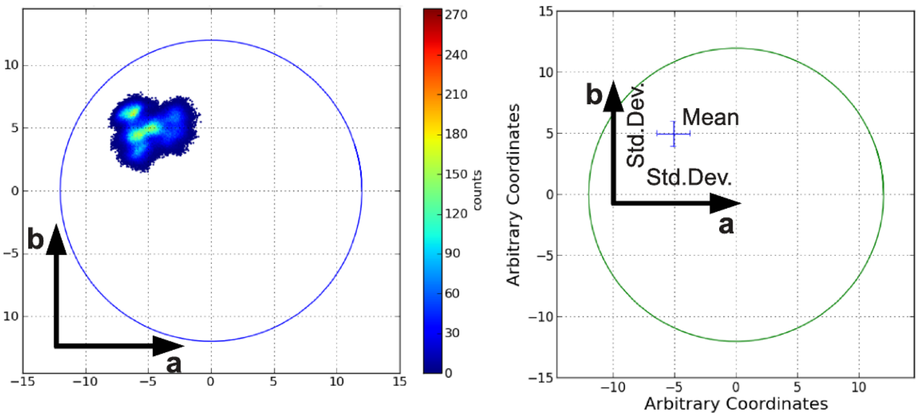

Figure S10: **Ilustration for the cartesian marginalization of the ROMs presented in Table S5.** Mean and standard deviation of the ROM distribution function are decomposed in the cartesian coordinates of the projected plane.

Table S5: **Mean and standard deviation of the ROMs for dimers in the POD+CC aggregate in cartesian coordinates.** Inner circle (ic) dimers are of type AB, outer circle (oc) dimers of type CC. The corresponding ROMs are plotted in Figs. 7 and 8 of the main text.

		Axis X		Axis Y		Axis Z	
		a	b	a	b	a	b
Atomistic ic	Mean	-5.0629	4.9695	5.3453	2.1628	0.1699	-7.6747
	Std.Dev.	1.3502	1.0551	0.9886	1.0650	1.4741	0.3541
Atomistic oc	Mean	-4.3698	4.3381	5.1197	2.4338	1.1021	-7.6593
	Std.Dev.	2.5217	1.2152	1.3281	2.2048	1.6164	0.8570
Coarse grained							
K200 ic	Mean	-7.0411	5.4833	3.5500	3.1683	-1.9514	-6.8707
	Std.Dev.	0.6332	0.5122	0.8076	0.5378	0.5321	0.4247
K200 oc	Mean	-4.8791	6.2357	4.3811	2.8479	-2.0415	-7.0463
	Std.Dev.	0.7523	0.6092	0.8243	1.2231	1.0504	0.5095
K500 ic	Mean	-6.5630	5.1461	4.5099	2.3816	-2.1200	-7.0358
	Std.Dev.	0.7203	0.4626	0.7937	0.5045	0.7054	0.3995
K500 oc	Mean	-5.0642	5.6978	4.5694	2.1297	-2.3696	-6.9783
	Std.Dev.	0.6599	0.8742	0.6481	0.7446	0.9727	0.4223
IDEN ic	Mean	-5.3913	4.8976	5.0381	2.3686	-0.3821	-6.6201
	Std.Dev.	1.5088	1.0948	1.1371	1.2775	1.0114	0.4255
IDEN oc	Mean	-3.6406	5.9506	4.3273	2.1856	-1.0299	-5.8907
	Std.Dev.	1.5422	1.3516	1.2101	2.3151	1.8936	0.8055
